# Aflatoxin contamination of maize and groundnut in Burundi: Distribution of contamination, identification of causal agents and potential biocontrol genotypes of *Aspergillus flavus*

**DOI:** 10.3389/fmicb.2023.1106543

**Published:** 2023-03-13

**Authors:** Gedeon Nsabiyumva, Charity K. Mutegi, John M. Wagacha, Asha B. Mohamed, Nancy K. Njeru, Privat Ndayihanzamaso, Marie Chantal Niyuhire, Joseph Atehnkeng, Emmanuel Njukwe, Kenneth A. Callicott, Peter J. Cotty, Alejandro Ortega-Beltran, Ranajit Bandyopadhyay

**Affiliations:** ^1^Institut des Sciences Agronomiques du Burundi (ISABU), Bujumbura, Burundi; ^2^International Institute of Tropical Agriculture (IITA), Nairobi, Kenya; ^3^School of Biological Sciences, University of Nairobi, Nairobi, Kenya; ^4^Kenya Agricultural and Livestock Research Organization (KALRO), Katumani, Nairobi, Kenya; ^5^IITA, Bukavu, Democratic Republic of Congo; ^6^IITA, Bujumbura, Burundi; ^7^United States Department of Agriculture, Agricultural Research Service, Tucson, AZ, United States; ^8^College of Food Science and Engineering, Ocean University of China, Qingdao, China; ^9^IITA, Ibadan, Nigeria

**Keywords:** atoxigenic, biocontrol, toxigenic, *Aspergillus* section *Flavi*, diversity

## Abstract

Aflatoxin contamination of the staples maize and groundnut is a concern for health and economic impacts across sub-Saharan Africa. The current study (i) determined aflatoxin levels in maize and groundnut collected at harvest in Burundi, (ii) characterized populations of *Aspergillus* section *Flavi* associated with the two crops, and (iii) assessed aflatoxin-producing potentials among the recovered fungi. A total of 120 groundnut and 380 maize samples were collected at harvest from eight and 16 provinces, respectively. Most of the groundnut (93%) and maize (87%) contained aflatoxin below the European Union threshold, 4 μg/kg. Morphological characterization of the recovered *Aspergillus* section *Flavi* fungi revealed that the L-morphotype of *A. flavus* was the predominant species. Aflatoxin production potentials of the L-morphotype isolates were evaluated in maize fermentations. Some isolates produced over 137,000 μg/kg aflatoxin B_1_. Thus, despite the relatively low aflatoxin levels at harvest, the association of both crops with highly toxigenic fungi poses significant risk of post-harvest aflatoxin contamination and suggests measures to mitigate aflatoxin contamination in Burundi should be developed. Over 55% of the L-morphotype *A. flavus* did not produce aflatoxins. These atoxigenic L-morphotype fungi were characterized using molecular markers. Several atoxigenic genotypes were detected across the country and could be used as biocontrol agents. The results from the current study hold promise for developing aflatoxin management strategies centered on biocontrol for use in Burundi to reduce aflatoxin contamination throughout the value chain.

## 1. Introduction

Production of food crops including maize and groundnut in sub-Saharan Africa (SSA) faces a combination of challenges that reduce yields, including drought, pests, diseases, soil nutrient limitation, shortage of farm inputs and credit, climate change, low labor productivity, and high population pressure on farmland (Keya and Rubaihayo, 2013; Ludgate and Tata, 2015). Additionally, many crops are prone to contamination with aflatoxins, highly toxic secondary metabolites produced by *Aspergillus* section *Flavi* fungi (Pildain et al., 2008; Probst et al., 2010; Mutegi et al., 2012). Crop contamination with aflatoxins results in health and economic constraints in many regions (Probst et al., 2012). Also, the toxins increase mortality and reduce productivity in livestock (Ezekiel et al., 2014; Monson et al., 2015). Trade within and between countries is affected by the aflatoxin menace, leading to rejection of consignments, extra costs for product testing, and reduced marketable volumes (Ramesh et al., 2003; PACA, 2012; The Standard, 2019; Daily Nation, 2020).

There are four major aflatoxins: B_1_, B_2_, G_1_, and G_2_. Aflatoxin B_1_ is the most prevalent and toxic. *Aspergillus flavus* and *A. parasiticus* are the species most implicated in contamination events (Probst et al., 2007; Amaike and Keller, 2011). *A. flavus* produces B aflatoxins while *A. parasiticus* produces both B and G aflatoxins (Klich, 2007). *A. flavus* is subdivided in two morphotypes, L and S, which differ in genetic, physiological, and toxigenic characteristics (Cotty, 1989). Across SSA there are many types of aflatoxigenic fungi, several of them not formally described, that resemble the S-morphotype but that are not closely related (Frisvad et al., 2019; Singh et al., 2020). Some of those types of fungi produce B and G aflatoxins (Cotty and Cardwell, 1999). In the current paper, we use the term ‘fungi with S-morphology’ for all those *Aspergillus* resembling the S-morphotype of *A. flavus*, regardless of which aflatoxins they produce.

Demand for maize, an important aflatoxin-prone staple in Burundi, has been increasing due to rapid population growth. Maize production in the country reached over 260,000 tons in 2020 making it the sixth-most important crop after cassava, bananas, sweet potato, beans, and potato (Food and Agriculture Organization of the United Nations, 2022). Maize in Burundi is mainly grown by smallholder farmers (around 0.5 ha/farm) for household consumption (Keya and Rubaihayo, 2013; Ludgate and Tata, 2015). Groundnut, with an annual production of approximately 9,300 tons in Burundi (Food and Agriculture Organization of the United Nations, 2022), is a traditional food mainly consumed as a snack and as a constituent of salads, porridges, and soups. Vulnerability of crops to mycotoxins varies as do their distribution in various crop matrices.

Aflatoxin contamination can start in the field during crop development where it sometimes reaches dangerous levels (Mahuku et al., 2019). However, even when aflatoxin contamination is low at harvest, it can increase to dangerous levels under suboptimal storage (Seetha et al., 2017). In Burundi, aflatoxin content of maize and groundnut at harvest has not been carefully quantified. After harvest, farmers store maize and groundnut in their houses in pots, bags or spread on the floor. In the absence of drying, some such storage conditions can encourage growth of toxigenic fungi and subsequent mycotoxin production. Udomkun et al. (2018) reported high aflatoxin levels in cassava, maize and groundnut foodstuffs collected from local markets in Burundi. Sixty-eight per cent of the 300 collected samples (15% cassava, 77% maize and 84% groundnut) contained aflatoxin levels above the European Union Commission allowable threshold (EUC; 4 μg/kg; sum of aflatoxin B_1_, B_2_, G_1_, and G_2_) and 37% samples (39% maize and 51% groundnut) exceeded the East African Community (EAC) standard (10 μg/kg total aflatoxins). It is not known whether those levels were due to either pre- or post-harvest contamination, or a combination of both.

To better design appropriate aflatoxin management strategies in Burundi, the current study sought to (i) quantify aflatoxin levels in maize and groundnut collected at harvest across Burundi, (ii) characterize populations of *Aspergillus* section *Flavi* associated with the crops, (iii) quantify potentials of the recovered fungi to produce aflatoxins, and (iv) characterize molecularly the atoxigenic fungi. The obtained results provide knowledge on the extent of aflatoxin contamination in maize and groundnut in Burundi at harvest, as well as, the fungi responsible for aflatoxin contamination. A large number of atoxigenic fungi were detected, which can be put to use as components of aflatoxin management strategies across Burundi.

## 2. Materials and methods

### 2.1. Description of the study area

Burundi lies between latitudes 2.3°S to 4.5°S and longitudes 28.8°E to 31.0°E. The elevation across the country ranges between 770 and 2,670 m above sea level (DFID, 2009). The country has five agro-ecological zones (AEZ), which are described in Table 1. The highest average annual rainfall occurs in the Congo–Nile ridge while the lowest occurs in the East and Northern depressions (Niyongabo, 2008). Most of the topography in Burundi is hilly, which constrains cultivation practices due to soil erosion and makes mechanization difficult. There are two main cropping seasons in Burundi: a short-rain season from September to February and a long-rain season from February to May (DFID, 2009). Smallholder farmers produce maize and groundnut mainly during the short-rain season in Burundi (Collins et al., 2013).

**Table 1:** Characteristics of the five agro-ecological zones (AEZs) in Burundi.

AEZ	Percentage of total area	Elevation (m)	Average annual temperature (°C)	Average annual rainfall (mm)	Provinces within AEZ
The plain of Imbo	7	774–1,000	23	800–1,000	Makamba, Bururi, Bujumbura, Bubanza, Cibitoke
The west slope of Congo-Nile ridge	10	1,000–2,000	17–23	1,100–1,800	Bururi, Bujumbura, Bubanza, Cibitoke
The Congo-Nile ridge	15	2,000–2,670	12–16	1,500–2,000	Makamba, Bururi, Gitega, Mwaro, Bujumbura, Muramvya, Kayanza, Bubanza, Cibitoke
Central plateau	44	1,500–2,000	16–18	1,150–1,500	Gitega, Mwaro, Karuzi, Muramvya, Kayanza, Rutana, Ruyigi, Gitega, Karuzi, Cankuzo, Ngozi, Muyinga, Kirundo
The East and Northern depressions	24	1,320–1,500	20	600–1,100	Makamba, Rutana, Ruyigi, Cancuzo, Kirundo, Muyinga

Source: Niyongabo (2008).

### 2.2. Sampling and sample preparation

**Figure 1:**
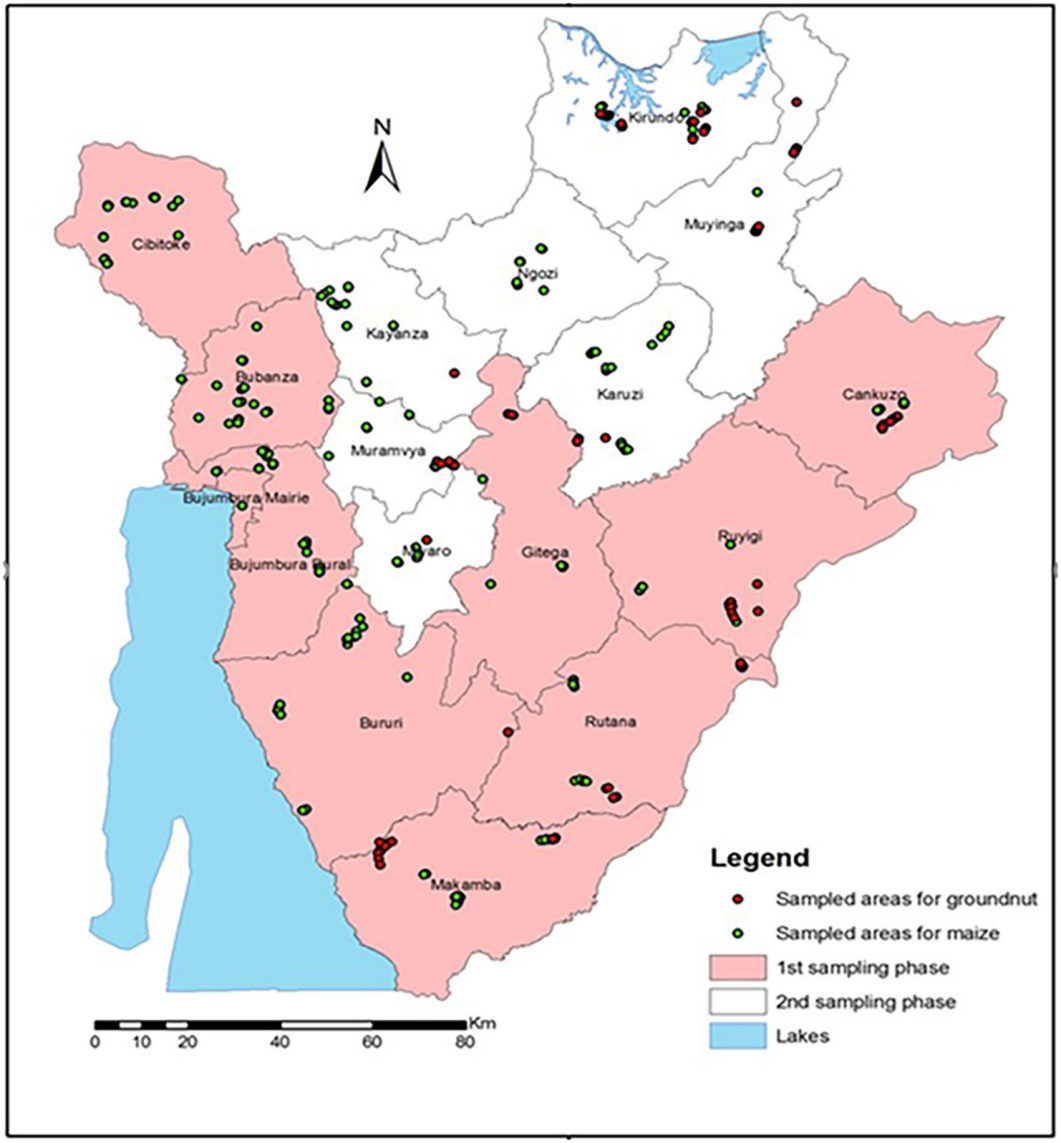
Maize and groundnut sampling sites in Burundi provinces in March and July 2014.

Sampling was conducted across the major maize and groundnut production areas of Burundi (Figure 1). Maize was sampled from 2–3 communes (in parenthesis) in all 16 provinces: Bubanza (Gihanga, Mpanda), Bujumbura (Mutimbuzi, Nyabiraba, Mukike), Bururi (Burambi, Rumonge, Mugamba), Cankuzo (Cankuzo, Gisagara), Cibitoke (Rugombo, Mabayi, Mugina), Gitega (Makebuko, Mutaho), Karuzi (Buhiga, Nyabikere, Bugenyuzi), Kayanza (Gatara, Matongo, Muruta), Kirundo (Busoni, Kirundo), Makamba (Kayogoro, Mabanda, Vugizo), Muramvya (Bukeye, Rutegama), Muyinga (Giteranyi, Muyinga), Mwaro (Kayokwe, Rusaka), Ngozi (Gashikanwa, Kiremba), Rutana (Bukemba, Musongati), and Ruyigi (Butaganzwa, Kinyinya). Groundnut was sampled from 8 provinces: Makamba, Rutana, Muyinga, Kirundo, Ruyigi, Cankuzo, Gitega, and Muramwa.

A total of 120 groundnut and 380 maize samples were collected from farmers’ fields at harvest, in two batches. The first batch (250) was collected in the lowlands and midlands in March 2014, after the end of the short-rain season. The samples were transported to KALRO Regional Mycotoxin Laboratory in Katumani, Kenya for processing. The second batch of samples (250) were harvested in highland areas in July 2014 at the end of the long-rain season and sent to Katumani for processing and analysis. Up to 10 maize cobs (~1 kg of grains after shelling) were sampled per field while groundnut pods were collected from up to 10 points in each farm (approximately 500 g when shelled). All samples were collected by moving in a zigzag line across each farm.

All cob/pod samples were sundried for 7 d, shelled manually and grain dried in a hot air oven (Memmert, United Kingdom) at 45°C for 48 h, to reach a moisture content of ≤ 13% for maize and ≤ 10% for groundnut. Moisture content was assessed using an Infratec™ 1241 Grain Analyzer (Foss, Denmark). Then, maize grains were ground using a coffee mill (Bunn-O-Matic Corp., Springfield, IL, United States) and groundnut grains were milled using a blender (BL335, Kenwood Intl., China). Both the coffee mill and blender were washed with 70% ethanol between samples to prevent cross contamination. All samples were divided in two halves, one for microbial and the other for aflatoxin analyzes. Halves for microbial analyzes were stored in hermetic bags at 4°C while those for aflatoxin analyzes were stored at −20°C until analysis.

### 2.3. Aflatoxin quantification in maize and groundnut

Aflatoxin levels in maize and groundnut were determined using Reveal Q+ for Aflatoxin kits and Accuscan Pro Reader (Neogen Corp., Lansing, MI, United States) following the manufacturer’s instructions. After homogenizing the sub-samples, 10 g were taken, mixed with 50 ml 65% ethanol, and shaken for 3 min using an orbital shaker (HS501 IKA-WERKE, Germany). The mixture was filtered through fluted Whatman No. 4 filter paper (Whatman Intl. Ltd., Maidstone, England) into a Tri-Pour^®^ beaker. Thereafter, 500 μL sample diluent was transferred to a sample cup and 100 μL of sample filtrate was added and mixed by pipetting up and down seven times. A 100-μL of diluted sample extract was transferred into a new sample cup. A strip was placed into the sample cup and left for 6 min. Then, the strip was read in the Accuscan Pro Reader. The lower detection limit of the Reader was 2 μg/kg while the upper detection limit was 150 μg/kg. Samples with more than 150 μg/kg were serially diluted in 65% ethanol, re-analyzed and the dilution factor was considered during the interpretation of results.

### 2.4. Isolation and identification of *Aspergillus* section *Flavi* from maize and groundnut

For microbiological analyzes, the sub-samples from batch 1 were sent to IITA Pathology and Mycotoxin Laboratory in Ibadan, Nigeria under appropriate import/export permits provided by phytosanitary authorities. The sub-samples from batch 2 were analyzed in Katumani. *Aspergillus* section *Flavi* were isolated and identified as in previous studies in our research group (Atehnkeng et al., 2008; Agbetiameh et al., 2018). Modified Rose Bengal Agar (MRBA; 3 g sucrose, 3 g NaNO_3_, 0.75 g KH_2_PO_4_, 0.25 g K_2_HPO_4_, 0.5 g MgSO_4_.7H_2_O, 0.5 g KCl, 10 g NaCl, 1 mL A&M micronutrients, 0.025 g Rose Bengal stock solution, 0.05 g chloramphenicol, 1 L distilled water, pH = 6.5) was used for isolation while 5–2 agar (50 mL V-8™ juice, 950 mL distilled water, 20 g agar, pH = 6.0) was used for both identifying *Aspergillus* section *Flavi* fungi as well as for saving sporulating cultures. Both media were autoclaved for 20 min at 121°C and cooled to <60°C. After cooling, 0.01 g dichloran and 0.05 g streptomycin sulfate were added to MRBA before pouring into Petri dishes.

Briefly, 1 g of each maize and groundnut sample was obtained from a thoroughly mixed sub-sample and suspended in 10 mL sterile distilled water. The suspension was homogenized by vortexing for 30 s and aliquots of 100 μL were inoculated on MRBA in a biosafety cabinet. Inoculated plates were incubated in the dark (31°C, 3 d). Putative *Aspergillus* section *Flavi* colonies were transferred to 5–2 plates using sterile toothpicks. When the number of putative section *Flavi* colonies per plate exceeded 10, the sub-sample was serially diluted and re-plated. When section *Flavi* colonies were not detected in a sample, the sub-sample weight or aliquot was increased accordingly. Colony forming units (CFU) of *Aspergillus* section *Flavi* per g of maize and groundnut were calculated as follows: CFU/g = (number of colonies × dilution factor) / weight of samples.

Isolates with greenish-yellow colonies and no or large sclerotia were identified as *A. flavus* L-morphotype. Isolates with numerous small sclerotia were classified as fungi with S-morphology. Colonies showing dark green color and large, rough dark-green spores were assigned as *A. parasiticus*. Colonies showing brown color were identified as *A. tamarii*. Pure cultures of 12 isolates per sample were stored in 4 mL vials containing 2 mL sterile water and stored at room temperature (23 ± 2°C) for short-term storage. For long-term storage, cultures were stored on silica gel at 4°C.

### 2.5. Determination of aflatoxin production ability of *Aspergillus* section *Flavi in vitro*

#### 2.5.1. Inoculation of *Aspergillus* section *Flavi* isolates in aflatoxin-free maize

Aflatoxin-free maize grains were sourced in both Katumani and Ibadan. Maize samples were analyzed using Accuscan Pro as above and considered as aflatoxin-free when no aflatoxin was detected in five tests. Five grams of aflatoxin-free grains were weighed into 40 mL clear glass vials, washed with tap water, and soaked overnight in 20 mL distilled water to adjust moisture content to 25%. The grains were then washed thrice with tap water to remove any fermentation product and thereafter autoclaved (20 min, 121°C, 15 psi). The sterile grains were independently inoculated with 500-μL spore suspension (approximately 10^6^ spores/mL) of an *Aspergillus* section *Flavi* isolate. Cultures were incubated at 31°C (dark, 7 d) for fermentation of maize grains by the inoculated isolates. Vials containing sterile grains inoculated with sterile water were used as controls.

#### 2.5.2. Extraction and quantification of aflatoxin from maize fermentations

Methodologies reported by Atehnkeng et al. (2008) and Ezekiel et al. (2014) were used to extract and quantify aflatoxins. After incubation, maize fermentations were stopped by adding 50 mL 70% methanol and the colonized grains were ground for 3 min using a high-speed blender (Waring commercial, Springfield, IL, United States). The mixture was transferred to a 250 mL separating funnel and 25 mL distilled water added. Thereafter, aflatoxin was partitioned twice by adding 6.25 mL dichloromethane followed by 2.5 mL dichloromethane. The dichloromethane extracts were passed through a bed of anhydrous sodium sulfate contained in fluted Whatman No. 4 filter paper into a Tri-Pour beaker and evaporated to dryness in the dark in a fume hood. The dried extract was dissolved in 1 mL dichloromethane and poured into a 1.5 mL Eppendorf tube. The toxin was then evaporated to dryness and tubes were stored at 4°C in darkness. Extracts were sent to IITA-Ibadan *via* courier for quantification.

In Ibadan, aflatoxin extracts were re-dissolved in 1 mL dichloromethane. Then, extracts and aflatoxin standards of known concentrations were spotted on thin-layer chromatography (TLC) plates. The plates were developed in diethylene:methanol:water (96:3:1) solution and then visualized under UV light (365 nm). The presence or absence of aflatoxin B_1_, B_2_, G_1_, and G_2_ was scored visually. Then, aflatoxins were quantified using a TLC Scanner 3 (CAMAG, Muttenz, Switzerland) with winCATS 1.4.2 software (Camag AG, Muttenz, Switzerland). The limit of quantification for all experiments was 2 μg/kg.

### 2.6. Microsatellite genotyping of atoxigenic fungi

A total of 1,335 *A. flavus* L-morphotype isolates that did not produce aflatoxins in maize fermentations (1,167 from maize and 168 from groundnut) were characterized using simple sequence repeats (SSRs) developed for *A. flavus* (Grubisha and Cotty, 2009). Previously described protocols were used to extract DNA from single-spored isolates and conduct the multiplex-PCR and microsatellite genotyping analyzes (Callicott and Cotty, 2015; Islam et al., 2018).

### 2.7. Laboratory competition assay

The ability of representative isolates of 11 selected atoxigenic SSR haplotypes to reduce aflatoxin when co-inoculated with a potent aflatoxin-producing *A. flavus* isolate (BUM009–08 from Burundi) was determined in laboratory competition assays as described earlier (Probst and Cotty, 2012; Agbetiameh et al., 2019). Briefly, inocula of single-spored isolates were grown on 5–2 agar. An equal amount of spore suspensions (1 × 10^6^ spores/mL) of individual atoxigenic isolate and the common toxigenic isolate (1 mL each) were combined and inoculated on 10 g autoclaved maize grain. Maize grains inoculated individually with each atoxigenic isolate, the toxigenic isolate, and water served as controls. There were four replications for each co-inoculation and control treatment. Following inoculation, protocols for maize fermentation and aflatoxin quantification process were similar as described in the previous sections.

### 2.8. Data analysis

Data on CFU/g, frequency of *Aspergillus* section *Flavi* species, and aflatoxins produced by the recovered fungi were analyzed using a negative binomial generalized linear model in R studio v3.5.3. Means were separated using Fisher’s protected least significance difference (LSD; α = 0.05). *Aspergillus* section *Flavi* isolates were categorized into aflatoxigenic and atoxigenic based on their ability to produce aflatoxin in maize fermentations. Aflatoxin content in samples at harvest was categorized into four levels: (i) aflatoxin below LOD of the kits (no aflatoxin), (ii) aflatoxin below the EUC threshold (4 μg/kg), (iii) aflatoxin above 4 μg/kg but below the EAC threshold (10 μg/kg), and (iv) aflatoxin above the EAC threshold. A chi-square test of association between aflatoxin concentrations and sample type (maize or groundnut) was performed in SPSS v.22 (IBM Corp, New York, United States). Correlation analysis among the population of *Aspergillus* section *Flavi* and levels of aflatoxins produced by selected isolates was performed in SPSS v.22.

Before analysis of SSR data, amplicon sizes were converted to repeat number by subtracting the size of the flanking region from the total amplicon size and then dividing by the size of the repeat. Allele frequencies and haplotypes were assessed with GenoDive (Meirmans and Van Tienderen, 2004). Relationships among unique haplotypes were displayed with a Neighbor-Net network generated with SplitsTree4 (Huson and Bryant, 2006) based on chord distances calculated with GenoDive. After sample-correcting the data by removing duplicate, identical haplotypes found in the same sample, an AMOVA (analysis of molecular variance) was performed using Arlequin v3.5.2.2 (Excoffier and Lishcer, 2010) to examine genetic variation by province.

## 3. Results

### 3.1. Aflatoxin concentration in maize and groundnut at harvest

**Figure 2:**
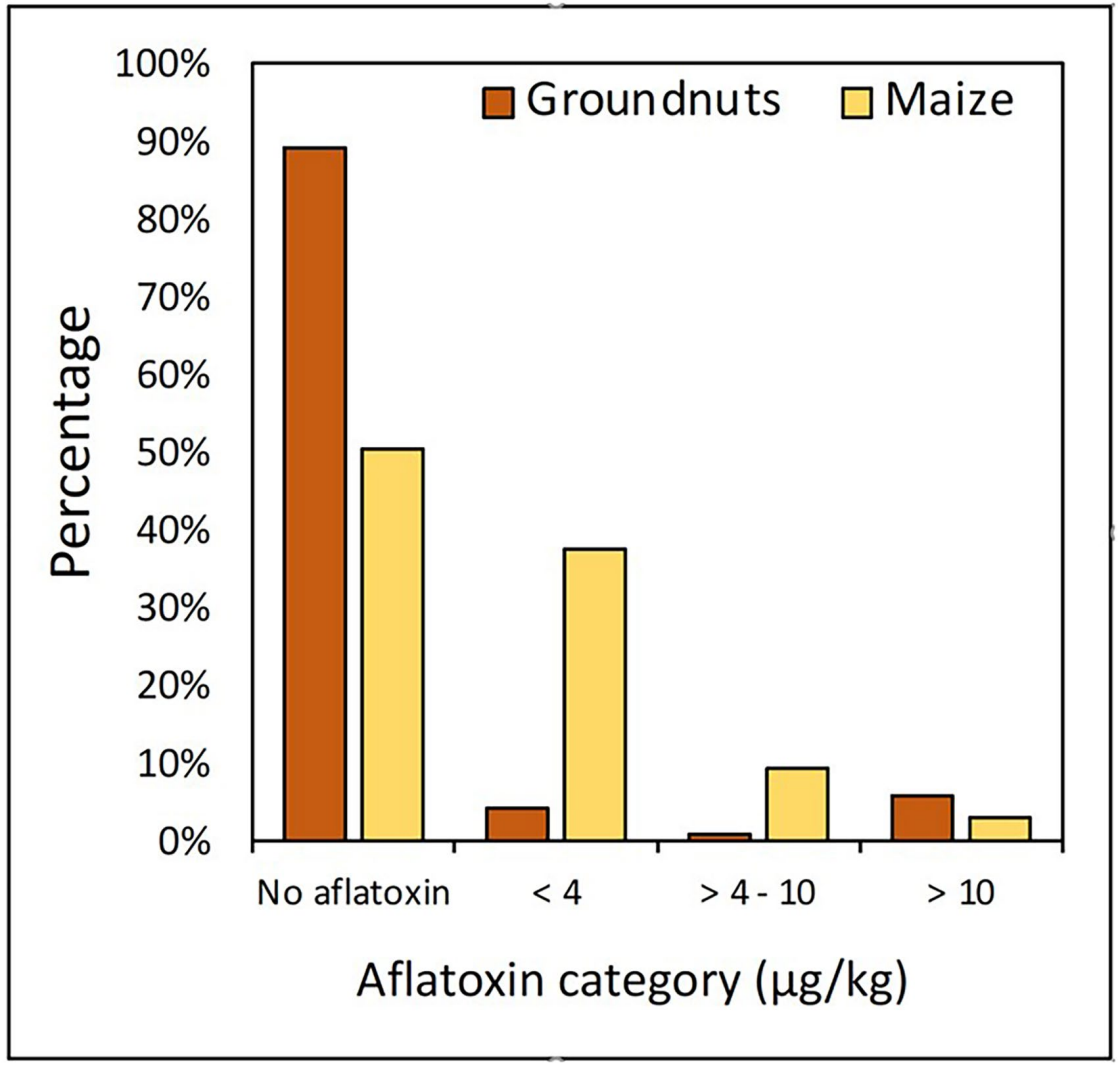
Proportion of maize and groundnut samples contaminated with different aflatoxin concentration categories.

Although 76% of both maize and groundnut contaminated with aflatoxin met the EUC threshold ( < 4 μg/kg), and a few maize (3%) and groundnut (6%) samples contained aflatoxin above the EAC threshold (10 μg/kg; Figure 2). Each of Burambi, Gihanga, Kayogoro, Kinyinya, Mpanda, Rugombo, and Kirundo communes had a maize sample contaminated with aflatoxin above 10 μg/kg, while Mabayi and Butaganzwa had two samples each. On the other hand, four groundnut samples (out of 10) from Vugizo had > 10 μg/kg aflatoxin compared to one sample each from Muyinga, Giteranyi, and Busoni.

### 3.2. Diversity of *Aspergillus* section *Flavi* in maize and groundnut

**Figure 3:**
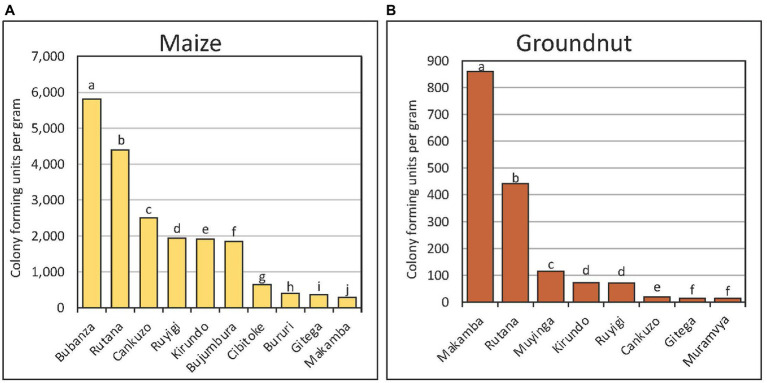
Population (colony forming units per gram) of *Aspergillus* section *Flavi* in maize **(A)** and groundnut **(B)** sampled from major provinces in Burundi. The letters attached to the bars represent statistical significance at 95% confidence level. *Aspergillus* population in maize was low in some provinces (Karuzi, Kayanza, Muramvya, Muyinga, Mwaro and Ngozo) and hence data for these provinces not plotted.

There were four types of fungi within *Aspergillus* section *Flavi* recovered from maize and groundnut: the *A. flavus* L-morphotype, fungi with S-morphology, *A. parasiticus*, and *A. tamarii*. The *Aspergillus* section *Flavi* population densities were significantly (*p* < 0.001) higher in maize (mean = 1,153 CFU/g) than in groundnut (mean = 266 CFU/g). For maize, there were significant (*p* < 0.001) differences in fungal densities among provinces (Figure 3A; data of only 10 of the 17 provinces shown) and within communes of a province (data not shown). The fungal density was low (< 50 CFU/g) in Karuzi, Kayanza, Muramvya, Muyinga, Mwaro, and Ngozi. Also, fungal densities in groundnut significantly (*p* < 0.005) differed among provinces (Figure 3B).

**Figure 4:**
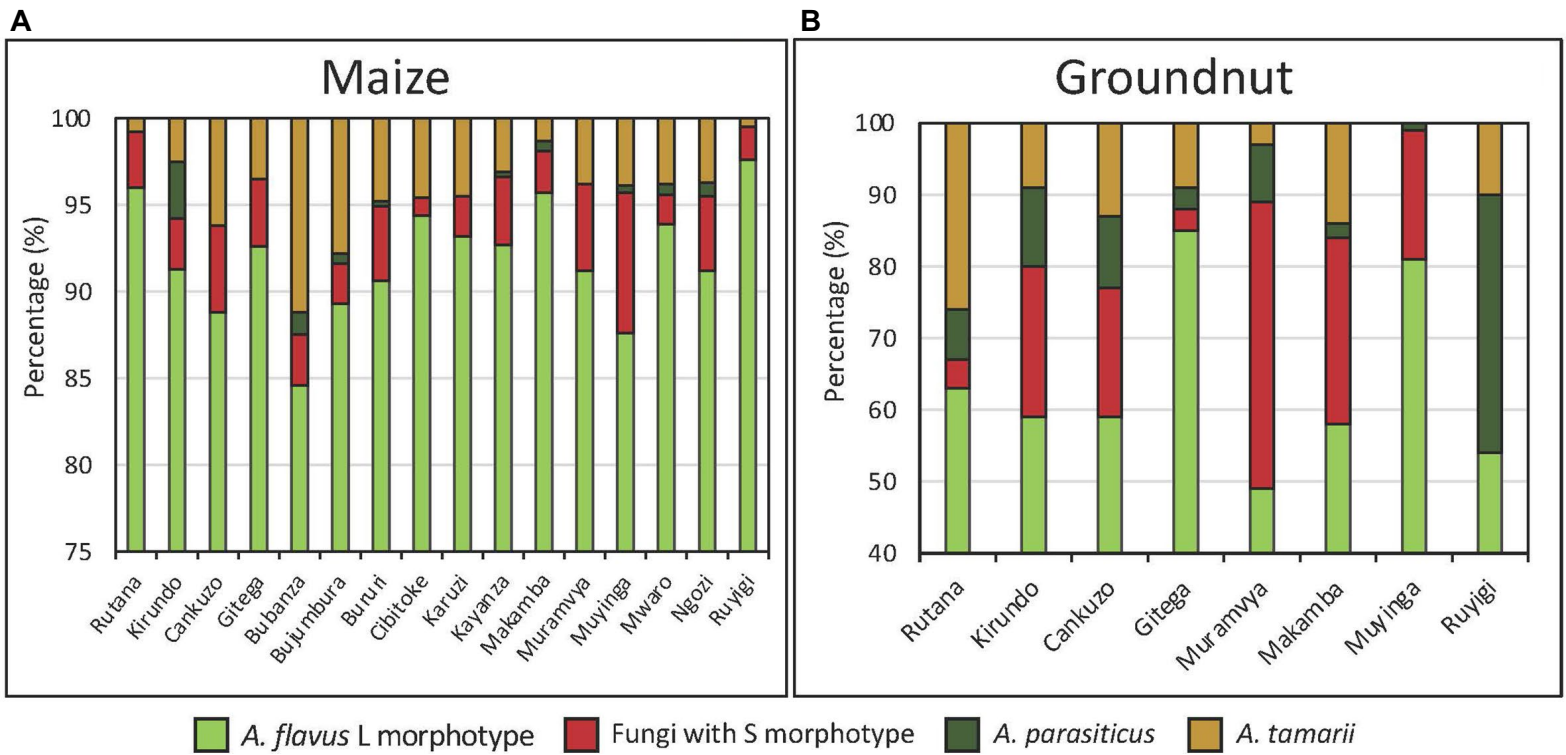
Proportion (%) of members of *Aspergillus* section *Flavi* recovered in maize **(A)** and groundnut **(B)** from major production provinces in Burundi.

The population of *Aspergillus* section *Flavi* in both maize and groundnut was dominated by the *A. flavus* L-morphotype. However, on an average, the proportion was significantly (*p* < 0.001) higher in maize (92%) than in groundnut (60%; Figures 4A,B). Proportions of *Aspergillus* section *Flavi* fungi significantly (*p* < 0.001) differed among provinces for both crops, with L-morphotype ranging from 85 to 98% in maize, and 45 to 85% in groundnut.

### 3.3. Aflatoxin concentrations produced by *Aspergillus* section *Flavi* isolates *in vitro*

Overall, 44.4% of the *Aspergillus* section *Flavi* isolates were toxigenic (Table 2). Proportionally, there were more toxigenic fungi with S-morphology isolates while there were more atoxigenic *A. flavus* L-morphotype isolates (Table 2). The concentration of aflatoxin B_1_ was significantly (*p* < 0.001) higher in isolates recovered from groundnut (mean = 2,617 μg/kg) than in isolates from maize (mean = 1,728 μg/kg). Fungi with S-morphology generally produced higher concentrations of each type of aflatoxin than the other isolates; *A. parasiticus* produced higher concentrations of B_2_ in groundnut and of G_1_ in maize. Overall, 93.5% of the fungi with S-morphology produced aflatoxins (31.9% produced only B aflatoxins); only two isolates of *A. parasiticus* did not produce aflatoxins. The recovered *A. tamarii* fungi did not produce aflatoxin, as expected (Table 2).

**Table 2:** Proportion (%) of aflatoxigenic and atoxigenic fungi in four members of *Aspergillus* section *Flavi* recovered from maize and groundnuts in Burundi and aflatoxin-producing potential of the toxigenic fungi.

Crop	Fungus	No. of isolates	Aflatoxigenic (%)	Atoxigenic (%)	Aflatoxin concentration (μg/kg)				
					B_1_	B_2_	G_1_	G_2_	Total
Maize	*A. flavus* L-morphotype	2,060	43.3	56.7	578	23	–	–	601
	Fungi with S-morphology^a^	60	91.7	8.3	2,260	90	396	47	2,792
	*A. parasiticus*	18	88.9	11.1	1,031	20	707	33	1,792
	*A. tamarii*	108	0	100	–	–	–	–	0
Groundnut	*A. flavus* L-morphotype	387	56.6	43.4	537	24	-	-	561
	Fungi with S-morphology^b^	17	100	0	3,012	42	1,653	80	4,786
	*A. parasiticus*	67	100	0	1,767	71	579	88	2,505
	*A. tamarii*	138	0	100	–	–	–	–	–

a There were 20 fungi with S-morphology isolates from maize that produced only B aflatoxins.

b There were three fungi with S-morphology isolates from groundnut that produced only B aflatoxins.

### 3.4. Correlations among population of *Aspergillus* section *Flavi* and aflatoxins produced by selected isolates

Concentrations of aflatoxins produced by selected *A. flavus* L-morphotype isolates had a negative correlation with the population of *A. flavus* L-morphotype in the samples from which the isolates were recovered (*r* = −0.508, *p* < 0.001). On the contrary, aflatoxin concentrations produced by selected fungi with S-morphology had a positive correlation with the population of this type of fungi in samples from which the isolates were recovered (*r* = 0.589, *p* < 0.001). These correlations were consistent for the types of aflatoxins produced by the two types of fungi. Moreover, there was a positive correlation between aflatoxin G_1_ (*r* = 0.315, *p* < 0.001) and G_2_ (*r* = 0.258, *p* = 0.002) produced by *A. parasiticus* with the population of the fungus in the samples from which the isolates were recovered.

### 3.5. Genetic diversity among atoxigenic fungi

**Figure 5:**
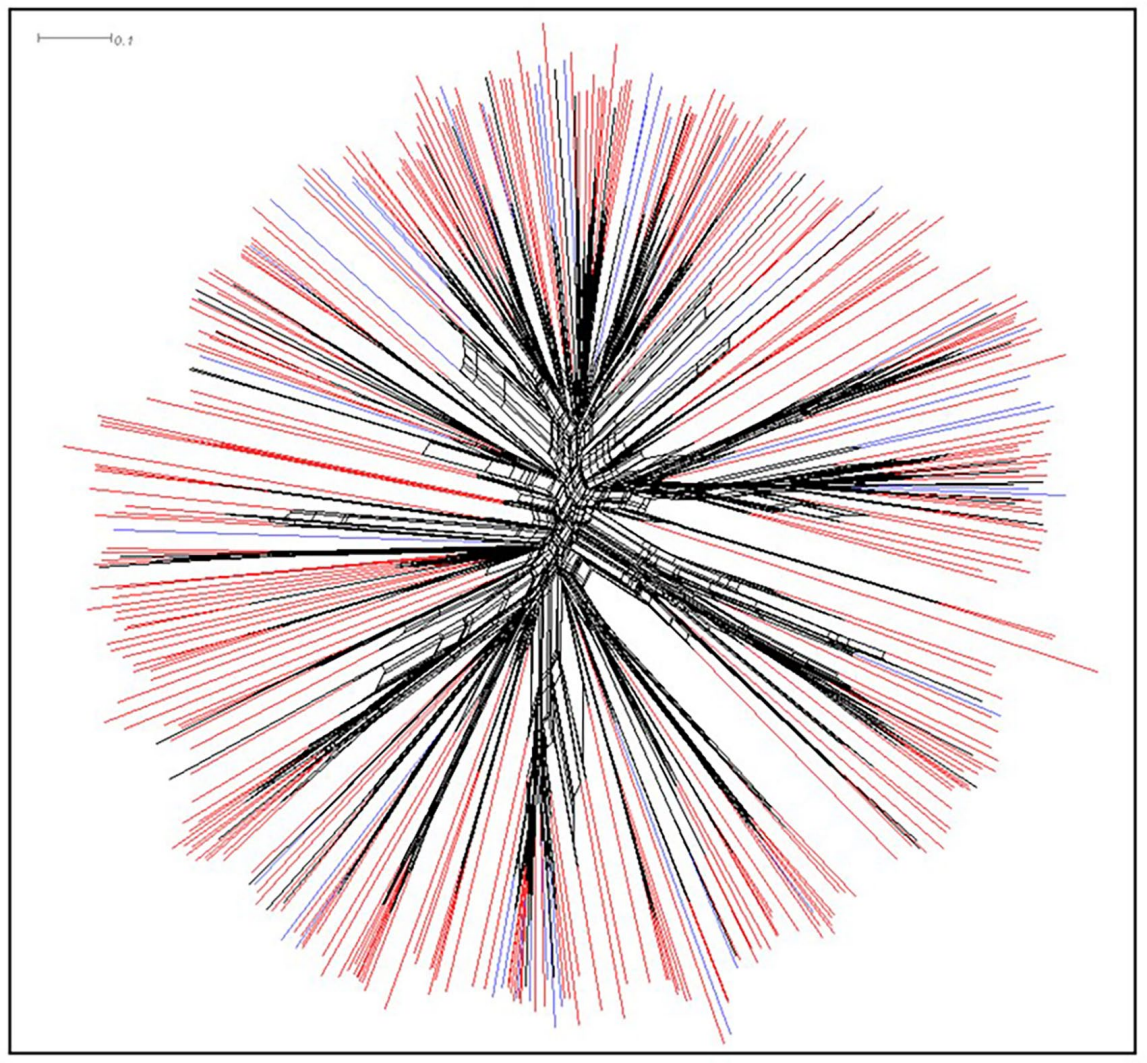
Neighbor-Net tree of 376 unique non-aflatoxigenic haplotypes found in Burundi. Haplotypes seen only in maize are shaded red, those found only in groundnut are shaded blue, and those in both crops are shaded black.

Atoxigenic isolates of *A. flavus* were highly diverse. There were 376 SSR haplotypes among the 1,335 atoxigenic isolates, representing 85 unique SSR haplotypes in the 168 groundnut isolates and 336 unique SSR haplotypes in 1,167 maize isolates. Supplementary Table S1 provides allele sizes of 17 SSR loci (Grubisha and Cotty, 2009) for the 376 SSR haplotypes of atoxigenic *A. flavus* found in Burundi. A Neighbor-Net visualization of the unique haplotypes (Figure 5) color coded by whether the haplotype was found in maize, groundnut, or both crops, shows a highly diverse collection of genotypes, with no separation by crop origin. A few groups of haplotypes were found only in maize (Figure 5). An AMOVA of isolates by crop origin confirmed this lack of separation, with 99.94% of the genetic variation found within each crop and only 0.06% between the two crops, with a fixation index close to zero (F_ST_ = 0.00063; *p* = 0.25; Supplementary Table S2). Likewise, no significant difference was seen among populations. For example, the 20 most frequent haplotypes were found in, on an average, 10 of the 17 provinces (Supplementary Table S3). This lack of differentiation is also seen using AMOVA; only 0.76% of the variation is attributable to differences among provinces, and as in the crop comparison, the fixation index is very low (F_ST_ = 0.0075; *p* = 0.00, Supplementary Table S4).

### 3.6. Laboratory competition assay

Representative isolates of the SSR haplotypes were evaluated for their ability to limit aflatoxin when co-inoculated with the highly toxigenic *A. flavus* isolate BUM009-08, which produced 4,480 μg/kg aflatoxins when inoculated alone (Table 3). Aflatoxin reduction ranged from 28.3 to 96.0%. Four isolates (BUG241-03, BUG208-07, BUM134-06, and BUM184-12) reduced aflatoxin by > 90%. Two more isolates (BUG242-04 and BUM033-05) had statistically similar, though numerically less, aflatoxin reductions compared to the four isolates named earlier. No aflatoxin was produced in maize grains inoculated with the atoxigenic isolates alone and in water control.

**Table 3:** Occurrence and distribution of selected atoxigenic haplotypes of *Aspergillus flavus* in Burundi and aflatoxin reduction by a representative isolate of the 11 haplotypes in maize grains co-inoculated with a toxigenic strains.

Atoxigenic isolate	Haplotype name	Number of isolates in the haplotype^a^	Number of samples^b^ with haplotype		Haplotype presence (√) or absence (×) within region					Total aflatoxin (μg/kg)	Aflatoxin reduction (%)^c^
			Maize	Groundnut	North	South	East	West	Center		
BUG241-03^d^	111AII	60	31	3	√	√	√	√	√	251	94.9 f
BUG208-07^d^	111AHG	62	38	3	√	√	√	√	√	242	90.6 ef
BUM033-05^d^	111AHH	30	17	5	√	√	×	√	√	1,342	71.2 cdef
BUM056-02^d^	111AHF	52	27	6	√	√	√	√	√	2,097	42.9 abc
BUM134-06	111AHU	28	13	1	×	√	×	√	√	123	94.7 f
BUM184-12	111AGX	26	8	6	×	√	√	√	√	241	96.0 f
BUG242-04	111AGO	14	9	2	×	√	√	√	√	1,563	73.7 def
BUM021-05	111AIA	35	26	6	×	√	√	√	√	1,241	62.9 bcde
BUM104-03	111AHS	12	7	3	×	√	√	√	×	2,514	55.1 abcd
BUG246-04	111AIB	37	19	6	√	√	√	√	√	1,971	33.7 ab
BUM115-12	111AHN	43	22	1	√	×	√	√	√	2,839	28.3 a
BUM009-08^e^	–^f^	–	–	–	–	–	–	–	–	4,480	–

a Out of 1,135 isolates characterized into 376 SSR haplotypes.

b Out of 380 maize samples and 120 groundnut samples.

c Percent total aflatoxin reduction was calculated as [1 – (aflatoxin content in maize co-inoculated with both toxigenic and atoxigenic isolate/aflatoxin content in maize inoculated with the aflatoxin-producing isolate alone)] × 100. Aflatoxin reduction values having a common letter are not significantly different according to Fischer’s Least Significant Difference (LSD) test (α = 0.05).

d Active ingredient of the biocontrol product Aflasafe BU01.

e BUM009-08 is an aflatoxin-producing isolate.

f Not determined.

### 3.7. Selection of genotypes with potential for use as aflatoxin biocontrol agents

**Figure 6:**
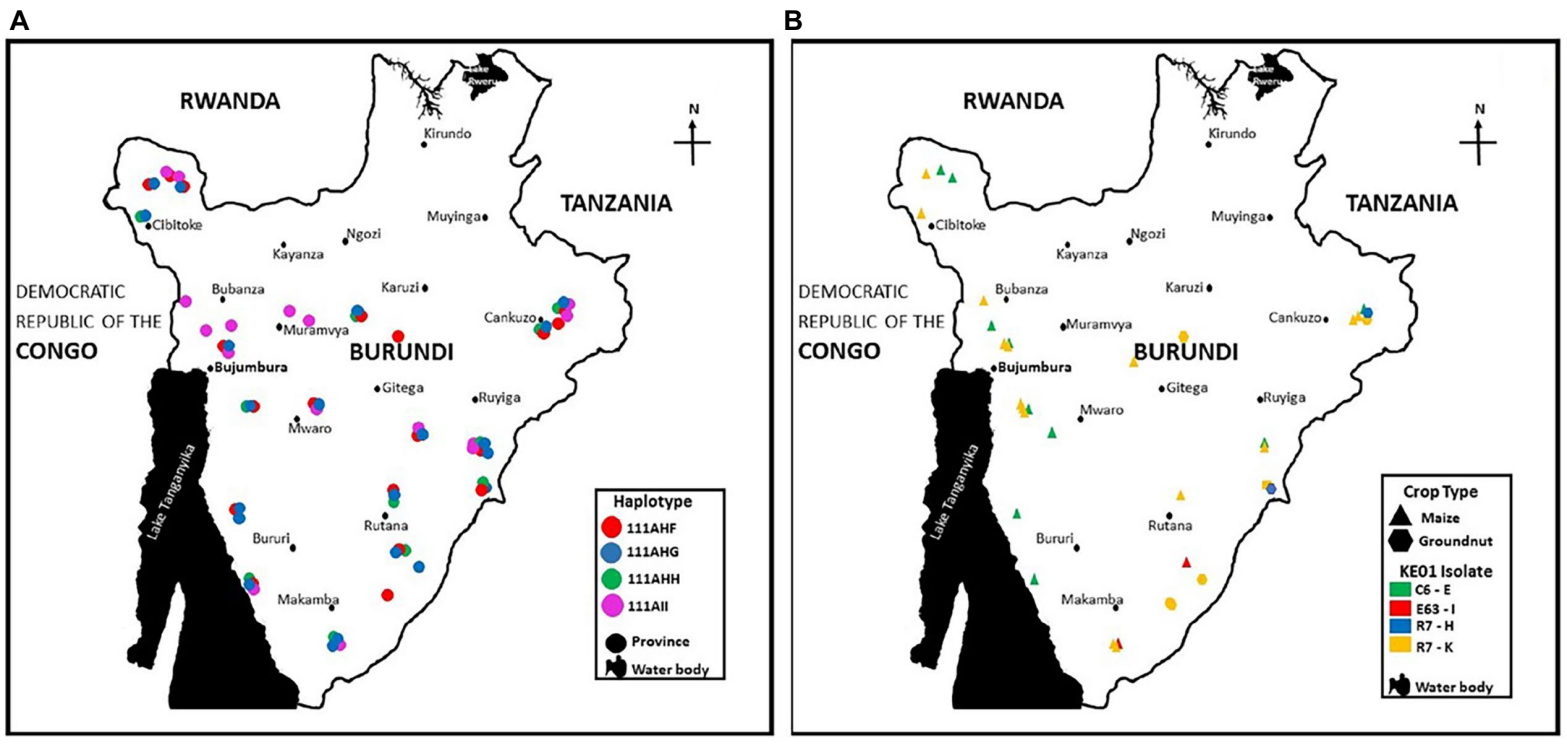
Distribution across Burundi of the active ingredient atoxigenic *Aspergillus flavus* isolates composing the aflatoxin biocontrol product Aflasafe BU01 **(A)** and Aflasafe KE01 **(B)**. Aflasafe KE01 was developed for use in Kenya but its active ingredients, apart from Burundi, have been detected in several other countries.

The population genetic analyzes revealed several atoxigenic SSR haplotypes widely distributed across Burundi (Figure 6). Some SSR haplotypes were found exclusively in maize or groundnut, while others were found in both crops (Figure 5). The distribution of haplotypes varied within five regions: north, south, center, east, and west (Table 3). Five haplotypes were detected in all five regions while other specific haplotypes were found in 1 to 4 regions. Wide distribution and large number of members were used as criteria for selection, in addition to their abilities to reduce aflatoxin in the co-inoculation. The four isolates selected as active ingredients of the biocontrol product Aflasafe BU01 (BUG241-03, BUG208-07, BUM033-05, and BUM056-02) had medium to high ability to limit aflatoxin contamination (Table 3). Each of the four belong to a unique SSR haplotype with frequent occurrence and wide distribution in at least 8 provinces located in 4 to 5 regions in Burundi (Figure 6A). Two other isolates (BUM184-12 and BUM021-05) were potentially good candidate active ingredients. Also, the analysis revealed that the genetic groups to which the active ingredient isolates of the biocontrol product Aflasafe KE01 belong to are also native to Burundi (Figure 6B; Supplementary Table S1), in addition to being common in Kenya, for which Aflasafe KE01 was originally developed. SSR fingerprints of the active ingredients of Aflasafe BU01 and Aflasafe KE01 are provided in Table 4.

**Table 4:** Allele sizes of 17 simple sequence repeat loci (AF28 to AF55; Grubisha and Cotty, 2009) for active ingredients of biocontrol products Aflasafe BU01 and Aflasafe KE01.

Biocontrol products	Active ingredient isolate	Haplotype	AF28	AF13	AF43	AF22	AF31	AF42	AF8	AF53	AF34	AF16	AF54	AF17	AF11	AF66	AF64	AF63	AF55
Aflasafe BU01	BUM056-02	111AHF	131^a^	148	385	192	352	159	171	134	301	169	161	359	123	261	169	127	180
	BUG208-07	111AHG	131	145	385	192	349	139	171	134	301	169	161	359	141	261	169	127	178
	BUM033-05	111AHH	119	164	399	144	312	143	168	131	296	169	161	371	138	269	163	127	172
	BUG241-03	111AII	113	148	393	184	370	162	218	188	314	178	161	374	162	275	180	131	176
Aflasafe KE01	R7K	H-1462	135	155	385	192	352	159	171	134	301	169	161	359	123	275	191	127	180
	C6E	H-0199	113	141	379	192	315	159	171	134	320	169	161	359	159	279	169	129	184
	E63-I	H-1017	131	135	379	196	361	181	215	134	301	169	172	359	123	255	169	133	178
	R7H	H-0212	113	141	379	196	361	181	209	134	320	178	172	359	159	253	227	129	178

a Allele sizes indicate amplicon size in base pairs as called on an ABI 3730 DNA Analyzer with the LIZ500 standard (Applied Biosystems). Islam et al. (2021) previously published data of Aflasafe KE01. However, the haplotype name and allele size of AF66 for active ingredient E63-I has been corrected in the Table.

## 4. Discussion

In the current study, maize and groundnut produced in Burundi were examined for aflatoxin content at harvest. Over 75% of the crops contained aflatoxin levels considered by the EUC as safe for human consumption (less than 4 μg/kg total aflatoxin). Both crops were associated with various types of aflatoxin-producing fungi, including some with the capacity to contaminate crops with dangerous concentrations of aflatoxins. The fungal communities were dominated by the *A. flavus* L-morphotype and most members of this group were atoxigenic. Other members of the communities were fungi with S-morphology and *A. parasiticus*; both of which exhibited high capacity to produce aflatoxins. Within atoxigenic fungi (1,335 isolates), several genotypes associated with both maize and groundnut were detected across Burundi, including genotypes of active ingredients of the aflatoxin biocontrol product Aflasafe KE01already registered for use in Kenya. The results from the current study provide evidence of the extent of aflatoxin contamination of maize and groundnut at harvest in Burundi and revealed structures and compositions of fungal communities associated with the two crops, with some members of those communities posing a risk during the postharvest stage while others with potential for use in aflatoxin management. The identified fungi with potential for use as biocontrol agents in Burundi need to be tested across Burundi to determine their effectiveness in limiting aflatoxin contamination at pre- and post-harvest stages (Bandyopadhyay et al., 2022).

Overall, only a small fraction of the samples contained aflatoxins above 4 μg/kg, the EUC threshold (Figure 2). Aflatoxins were not detected in most (approximately 90%) of the groundnut and maize (50%) samples. The environmental conditions of the five AEZs in Burundi (Table 1) may not allow for aflatoxin production in the field despite the association of both crops with aflatoxin-producers (Figure 3) and the high aflatoxin-production potential of a significant proportion of the associated fungi (Table 2). However, apart from potential aflatoxin contamination in the field, crops associated with potent aflatoxin producers are at high risk of contamination, if stored under sub-optimal conditions (Hell et al., 2008; Seetha et al., 2017; Senghor et al., 2020), or if climate change result in conditions favorable for aflatoxin production. Unfortunately, models indicate that in large portions of East Africa, including Burundi, maize production will become severally affected by climate change (Ojara et al., 2021). In Burundi, as in many other countries in SSA, storage needs significant improvement to discourage post-harvest losses, including aflatoxin contamination (Udomkun et al., 2017). Most maize and groundnut samples met the EUC threshold (<4 μg/kg) and hence were regarded as safe for human consumption. However, low aflatoxin levels at harvest do not necessarily mean that the crops will remain safe, especially in the absence of integrated programs promoting food safety from field to fork.

The *A. flavus* L-morphotype was the predominant species in both maize and groundnut (Figure 4). Similarly, the L-morphotype composes ~80% of the maize and groundnut communities in various SSA countries (Atehnkeng et al., 2008; Mutegi et al., 2012; Probst et al., 2012). Dominance of the L-morphotype significantly corresponded with low levels of aflatoxin in most maize and groundnut, which concurs with findings of a study in Kenya reporting that the L-morphotype dominated non-aflatoxin-outbreak regions while fungi with S-morphology dominated aflatoxin outbreak regions (Probst et al., 2010). On the other hand, communities of the *A. flavus* L-morphotype have been reported to produce high aflatoxin levels (Atehnkeng et al., 2008; Okoth et al., 2012; Agbetiameh et al., 2019) and therefore high prevalence of the L-morphotype cannot be used as a conclusive proxy for the aflatoxin concentration of a crop. The incidence of *A. parasiticus* was relatively low in both groundnut and maize. In other reports, *A. parasiticus* has been reported to have a high association with groundnut fields (Horn and Dorner, 1998; Kachapulula et al., 2017). However, several studies from our research group have noticed low incidence of *A. parasiticus* in groundnut samples in certain countries: Ghana (Agbetiameh et al., 2019), Mali and Sudan (unpublished). It is unclear which factor led to relatively low levels of *A. parasiticus* in groundnut in Burundi.

Around 40% of *A. flavus* L-morphotype were toxigenic (Table 2). Over 2 decades ago, the same percentage of *A. flavus* isolated from foods in Burundi were toxigenic (Munimbazi and Bullerman, 1996). In addition, over 90% of both fungi with S-morphology and *A. parasiticus* exhibited high aflatoxin production potential (Table 2), which is a norm for both groups of fungi. The high levels of aflatoxins (>1,500 μg/kg) in some groundnut samples can be attributed to the presence of potent toxigenic strains. The levels of aflatoxin produced *in vitro* by fungi with S-morphology and *A. parasiticus* had a positive correlation with the population of each type of fungi. Similar studies (Jaime-Garcia and Cotty, 2006; Probst et al., 2007, 2012; Mauro et al., 2015) showed a positive correlation between the presence of fungi with S-morphology and high aflatoxin levels. Since the examined crops were collected at harvest, there is still a high risk of contamination during storage and subsequent exposure of consumers of the foodstuffs, if aflatoxin-conducive conditions occur throughout storage and before consumption.

Fungi with S-morphology were more toxigenic than *A. flavus* L-morphotype, as previously reported (Probst et al., 2007; Mutegi et al., 2012). Therefore, even low levels of fungi with S-morphology are a high risk to accumulation of unsafe aflatoxin levels in crops. Fungi with S-morphology were implicated in the high levels of aflatoxin contamination that claimed more than 125 lives in lower eastern Kenya in 2004 (Probst et al., 2012, 2014). However, Probst et al. (2011) also reported that 33% of *A. flavus* L-morphotype isolates recovered from Kenya were not toxigenic. The high prevalence of atoxigenic *A. flavus* L-morphotype isolates is encouraging, as it provides many potential aflatoxin biocontrol agents to protect maize and groundnut (Probst et al., 2011; Agbetiameh et al., 2019). Indeed, when the atoxigenic isolates were genotyped with SSR markers, a few SSR genotypes occurred in high frequency and in several provinces demonstrating wide distribution and hence potential of high adaptation in the country (Table 3; Supplementary Table S1). An extensive *Aspergillus* population distribution study in Kenyan soil showed that the active ingredients of the biocontrol product Aflasafe KE01 are widely distributed in Kenya (Islam et al., 2021). Isolates of some representative Burundi-specific SSR groups detected in the current study were tested in laboratory assays as potential candidates for the development of biocontrol products for aflatoxin management for use in Burundi (Table 3).

There are several criteria for selecting atoxigenic isolates to constitute biocontrol products containing multiple active ingredients, including frequent occurrence and wide distribution across the target region, membership to VCGs composed entirely of atoxigenic members, superior ability to reduce aflatoxins in laboratory experiments (Moral et al., 2020) and displacement ability in field experiments (Agbetiameh et al., 2019), among others. Recently, our group reported that in some cases, after application, some active ingredient fungi are found at higher proportions in the soil while others in the grain (Atehnkeng et al., 2022). Those results suggest that under some conditions some active ingredient isolates may be better displacers of aflatoxin producers in the soil than the others, and those others may competitively displace better in the grain that would appear as the major contributor on aflatoxin reduction. However, the displacement in the soil is important to prevent aflatoxin producers from reaching the maturing crop.

For biocontrol formulation for Burundi, a balanced selection took into consideration isolates belonging to SSR groups with frequent occurrence and wide distribution, and high aflatoxin reductions. Therefore, there was one isolate (BUM056-02) from a group with frequent occurrence, detected in many regions across Burundi, but with relatively less aflatoxin reduction in the competition experiments (Table 3). We hypothesize that the isolate will compensate its relatively lower ability to reduce aflatoxin in the laboratory with its ability to dominate crops and soils from the target areas. Indeed, initial field testing of the biocontrol product developed for Burundi reveals that treated crops contain lower aflatoxin levels than nontreated crops (data not shown). Thus, the rationale for selecting the active ingredient fungi appears to be correct. Nonetheless, an advantage of characterizing large numbers of *A. flavus* isolates in Burundi is that a rich germplasm of atoxigenic *A. flavus* is available to replace any active ingredient isolate of Aflasafe BU01, if performing poorly in the soil and the crop. It has also been argued that aflatoxin biocontrol products containing various active ingredient fungi should contain isolates with opposing mating-type idiomorphs (Moore, 2022). Three of the selected isolates (BUG241-03, BUG208-07, and BUM056-02) contain the MAT1-1 idiomorph while the other (BUM033-05) contains the MAT1-2 idiomorph (data not shown).

In various SSA countries, use of aflatoxin biocontrol products based on atoxigenic fungi have been developed, tested, registered, and transferred to the private sector for large scale use (Probst et al., 2011; Agbetiameh et al., 2019; Bandyopadhyay et al., 2022). Atoxigenic fungi competitively exclude aflatoxin-producing *Aspergillus* from the crop environment, and this results in low aflatoxin levels in crops at harvest and during storage, even under sub-optimal conditions (Senghor et al., 2020). While there were groups of atoxigenic haplotypes only seen in maize, it is likely that this is due to the much larger maize sample size (Table 2). We detected atoxigenic fungal genotypes never reported outside of Burundi (Figure 6A), but also atoxigenic genotypes native to Kenya (Figure 6B) that have been already registered for use as active ingredients of the biocontrol product Aflasafe KE01 (Adhikari et al., 2016; Moral et al., 2020). Extension of the label for use of Aflasafe KE01 in groundnut and sorghum is ongoing.

East Africa has a broad diversity of agroecologies including both high elevation and low elevation production areas. Between Kenya and Burundi these agroecologies are well represented. Frequent association of the active ingredients of Aflasafe KE01 with maize and groundnut produced in Burundi, in addition to Uganda (G. Mahuku, personal communication) indicates that the Aflasafe KE01 active ingredients may be broadly effective across East Africa. On-going field testing of a country specific Aflasafe product for use in Burundi, Aflasafe BU01 (Figure 6A), and Aflasafe KE01 hold promise to manage aflatoxin contamination in Burundi. Both Burundi and Kenya belong to the EAC, which also includes Uganda, Rwanda, Tanzania, and DR Congo. EAC Partner States are promoting the use of biocontrol through ongoing harmonization of regional regulatory frameworks for biocontrol agents (Ortega-Beltran and Bandyopadhyay, 2021).^1^

## 5. Conclusion

Only a small proportion of samples (groundnut = 6%, maize = 3%) was contaminated with aflatoxins above the EAC threshold of 10 μg/kg. However, maize and groundnut were associated with highly toxigenic fungi, representing a risk of contamination during the post-harvest stages, which may last for more than 1 year. Further studies to assess contamination further up the value chain are necessary. The population of *Aspergillus* section *Flavi* in both maize and groundnut was composed of a significant proportion of aflatoxigenic strains. Fungi with S-morphology were the most toxigenic while *A. flavus* L-morphotype isolates were mostly atoxigenic. While the observed and potential contamination can be attributed to the fungi with S-morphology, the high proportion of *A. flavus* L-morphotype provides hope for developing biocontrol products for use in Burundi. Indeed, among the L-morphotype isolates of *A. flavus*, 55% were atoxigenic within which a few genetic groups were widely distributed in Burundi. The active ingredients of the biocontrol product registered for use in Kenya (Aflasafe KE01) were also found in Burundi and could be evaluated in Burundi. In addition, another biocontrol product, Aflasafe BU01, was formulated with four different widely distributed genetic groups specific to Burundi. However, biocontrol must be supported by other interventions including awareness creation, timely harvesting, rapid grain drying, appropriate storage structures, sorting, and processing and insect control at pre-and post-harvest stages.

## Data availability statement

The original contributions presented in the study are included in the article/Supplementary material, further inquiries can be directed to the corresponding author.

## Author contributions

RB, PC, and CM designed the overall projects from which data are derived. GN, CM, JW, PN, JA, EN, PC, and RB contributed to the conception and design of the experiments. GN, PN, MN, and EN collected the grain samples. GN, CM, JW, AM, NN, JA, and AO-B conducted and analyzed the microbiology and aflatoxin studies. KC and PC collected and analyzed the molecular data. AO-B, KC, and RB performed distribution mapping of the genetic groups. CM, PC, AO-B, and RB provided guidance. GN, CM, and AO-B drafted the original manuscript followed by editing by other authors. RB and PC secured funds for the study. All authors contributed to the article and approved the submitted version.

## Funding

The study was supported by the United States Agency for International Development (USAID), through the Aflatoxin Policy and Program for the East Africa Region (APPEAR) project that was implemented by the International Institute of Tropical Agriculture (IITA), Institut des Science Agronomiques du Burundi (ISABU), Kenya Agricultural and Livestock Research Organization (KALRO) and the East African Community. We acknowledge partial support from the Bill & Melinda Gate Foundation (OPP1133356). Support was also provided by USDA Agricultural Research Project 5347-42000-02-00D. We also gratefully acknowledge additional funding support from the CGIAR Research Program on Agriculture for Nutrition and Health (A4NH), and the CGIAR Plant Health Initiative by CGIAR Trust Fund contributors (https://www.cgiar.org/research/).

## Conflict of interest

The authors declare that the research was conducted in the absence of any commercial or financial relationships that could be construed as a potential conflict of interest.

## Publisher’s note

All claims expressed in this article are solely those of the authors and do not necessarily represent those of their affiliated organizations, or those of the publisher, the editors and the reviewers. Any product that may be evaluated in this article, or claim that may be made by its manufacturer, is not guaranteed or endorsed by the publisher.

## Author disclaimer

The use of trade, firm, or corporation names in these methods is for the information and convenience of the reader. Such use does not constitute an official endorsement or approval by the USDA Agricultural Research Service, of any product or service to the exclusion of others that may be suitable. In addition, USDA-ARS makes no warranties as to the merchantability or fitness of the methodologies described on these pages for any particular purpose, or any other warranties expressed or implied. These methodologies provide a guide and do not replace published work. USDA-ARS is not liable for any damages resulting from the use or misuse of these methodologies.

## Footnotes

^1^ https://www.eac.int/documents/category/aflatoxin-prevention-and-control
