# Supplementary material for: Aflatoxin contamination of maize and groundnut in Burundi: Distribution of contamination, identification of causal agents and potential biocontrol genotypes of Aspergillus flavus
Source: Front Microbiol. 2023 Mar 13;14:1106543. doi: 10.3389/fmicb.2023.1106543 (PMC10093718; doi:10.3389/fmicb.2023.1106543)
Supplement: Supplementary file 1 [file Table_1.pdf]

Supplementary Table 1. Allele sizes* of 17 simple sequence repeat loci (AF28 to AF55; Grubisha and Cotty 2009) for SSR haplotypes of atoxigenic *Aspergillus flavus* found in Burundi. Haplotypes belonging to the biocontrol product Aflasafe BU01 are in blue. Haplotypes belonging to Aflasafe KE01 are in bold and highlighted in yellow.

Haplotype	Groundnut isolates	Maize isolates	Provinces	AF28	AF13	AF43	AF22	AF31	AF53	AF34	AF42	AF8	AF16	AF54	AF17	AF11	AF66	AF64	AF63	AF55
111ABU / H-1017	7	34	12	131	135	379	196	361	134	301	181	215	169	172	359	123	255	169	133	178
111AGN	0	13	3	113	148	393	184	370	197	314	162	218	178	161	374	162	275	180	131	176
111AGO	2	12	5	113	141	385	184	367	154	310	174	168	175	161	359	153	271	215	131	180
111AGP	0	1	1	113	141	385	184	367	154	310	174	168	175	161	359	153	271	217	131	180
111AGQ	0	2	2	119	128	390	144	312	131	296	146	174	169	161	368	132	269	159	127	174
111AGR	0	1	1	119	138	408	184	358	163	314	159	233	172	161	374	159	277	183	131	176
111AGS / H-1462	13	80	15	135	155	385	192	352	134	301	159	171	169	161	359	123	275	191	127	180
111AGT	0	5	4	113	141	379	196	361	134	320	181	209	178	172	359	159	253	229	129	178
111AGU	0	8	4	113	145	387	192	367	134	301	159	171	169	161	359	141	273	169	127	178
111AGV	2	28	8	119	128	402	144	312	131	296	146	177	169	161	368	132	269	159	127	174
111AGW	2	19	8	131	135	379	196	337	134	301	181	212	169	172	359	123	255	169	133	178
111AGX	4	19	9	131	135	393	196	361	134	301	181	215	169	172	359	123	255	169	133	178
111AGY	0	1	1	113	145	379	192	322	134	301	159	171	169	161	350	159	261	183	137	182
111AGZ	0	2	1	113	158	370	192	325	134	301	181	160	169	161	353	138	263	183	127	184
111AHA	1	4	4	119	128	408	144	312	131	296	146	180	169	161	368	132	269	159	127	174
111AHB	3	16	8	113	158	387	192	315	134	342	159	160	172	161	353	138	263	183	127	178
111AHC	5	24	7	113	128	387	184	370	134	314	184	174	172	161	368	138	267	243	127	193
111AHD	0	1	1	119	141	414	184	352	298	317	159	242	169	161	377	132	277	211	131	207
111AHE	0	1	1	113	141	414	184	358	154	314	159	233	172	161	371	177	253	199	131	178
**111AHF**	7	45	10	131	148	385	192	352	134	301	159	171	169	161	359	123	261	169	127	180
**111AHG**	3	59	9	131	145	385	192	349	134	301	139	171	169	161	359	141	261	169	127	178
**111AHH**	5	25	9	119	164	399	144	312	131	296	143	168	169	161	371	138	269	163	127	172
111AHI	0	4	4	119	151	385	192	322	134	301	159	171	169	161	359	123	255	169	127	180

Haplotype	Groundnut isolates	Maize isolates	Provinces	AF28	AF13	AF43	AF22	AF31	AF53	AF34	AF42	AF8	AF16	AF54	AF17	AF11	AF66	AF64	AF63	AF55
111AHJ	0	1	1	113	141	373	184	358	154	314	162	183	172	161	371	177	253	199	131	178
111AHK	1	3	3	113	170	370	192	331	134	301	181	160	172	161	353	138	261	180	127	184
111AHL	6	15	11	119	148	405	144	312	131	296	146	147	169	161	362	123	269	161	127	178
111AHM	S3	40	11	131	141	385	196	322	134	301	156	171	175	161	359	141	261	221	127	178
111AHN	1	42	9	113	145	376	192	322	134	301	159	171	169	161	359	141	261	169	127	178
111AHO	6	17	11	113	141	414	184	358	154	314	162	183	172	161	371	177	255	199	131	178
111AHP	0	4	3	113	141	370	192	312	134	326	223	160	175	161	359	138	253	169	127	178
111AHQ	5	14	9	113	141	379	196	361	134	320	181	209	178	172	359	159	255	227	129	178
111AHR	0	1	1	113	138	396	184	364	144	314	159	183	175	161	377	174	255	180	131	182
111AHS	4	8	7	113	151	385	196	358	134	301	190	171	169	161	359	141	275	221	127	178
111AHT	0	12	4	113	122	385	184	337	134	317	205	206	172	161	359	135	267	183	127	197
111AHU	1	27	8	131	135	393	196	361	134	301	181	212	169	172	359	123	255	169	133	178
111AHV	1	2	3	113	148	385	184	373	154	317	181	230	172	161	353	138	271	279	127	191
111AHW	0	1	1	131	148	385	192	352	134	301	159	174	169	161	359	123	261	169	127	180
111AHX	1	1	2	119	164	399	144	312	131	304	143	168	169	161	371	138	269	163	127	172
111AHY	0	1	1	110	148	393	184	370	197	314	162	218	178	161	374	162	275	180	131	176
111AHZ	0	1	1	113	141	373	192	334	134	342	178	171	172	161	353	138	255	183	127	178
111AIA	6	29	11	113	141	414	184	358	154	314	162	183	172	161	371	177	253	199	131	178
111AIB	6	31	14	113	141	382	184	334	154	317	178	177	172	161	377	153	269	221	127	174
111AIC	0	2	1	113	141	385	176	349	154	317	159	197	172	161	362	144	273	203	127	199
111AID	0	1	1	113	148	393	184	370	197	314	162	215	178	161	374	162	275	180	131	176
111AIE	0	1	1	113	155	376	192	328	134	301	181	189	172	161	353	138	263	183	127	180
111AIF	0	1	1	113	148	393	184	370	197	314	162	218	178	161	374	165	275	180	131	176
111AIG	0	4	4	119	138	385	144	312	131	296	146	160	169	161	353	129	269	161	127	182
111AIH	0	1	1	131	122	379	184	337	151	301	208	194	172	161	353	141	267	174	127	186
**111AII**	3	57	8	113	148	393	184	370	188	314	162	218	178	161	374	162	275	180	131	176
111AIJ	1	1	2	131	135	379	196	361	134	301	181	212	169	172	359	123	255	169	133	178
111AIK	0	14	4	138	145	385	192	322	134	301	156	160	169	161	359	141	261	169	127	178
111AIL	0	1	1	113	151	393	196	343	134	301	159	171	169	161	359	141	275	215	127	178

Haplotype	Groundnut isolates	Maize isolates	Provinces	AF28	AF13	AF43	AF22	AF31	AF53	AF34	AF42	AF8	AF16	AF54	AF17	AF11	AF66	AF64	AF63	AF55
111AIM	0	1	1	135	145	385	192	346	134	301	184	160	169	161	356	141	261	169	127	180
111AIN	0	4	4	135	161	385	176	367	154	317	184	186	172	161	362	138	267	197	127	195
111AIO / H-0199	1	31	12	113	141	379	192	315	134	320	159	171	169	161	359	159	279	169	129	184
111AIP	0	2	2	119	128	414	144	312	131	296	146	174	169	161	368	132	269	159	127	174
111AIQ	0	1	1	110	161	385	184	334	151	314	165	191	175	161	377	156	277	178	131	178
111AIR	0	1	1	119	141	376	192	315	137	301	178	189	169	161	371	144	263	264	127	180
111AIS	0	6	3	113	161	385	184	334	151	314	165	191	175	161	377	156	277	178	131	178
111AIT	0	1	1	113	141	382	184	361	151	314	159	189	175	161	359	132	271	245	131	195
111AIU	0	1	1	113	151	393	184	331	163	317	187	200	172	161	374	174	267	243	127	180
111AIV / H-0212	7	22	12	113	141	379	196	361	134	320	181	209	178	172	359	159	253	227	129	178
111AIW	0	1	1	113	151	385	176	346	154	317	178	239	172	161	362	138	267	195	127	195
111AIX	0	5	4	113	141	382	184	334	154	317	178	177	172	161	377	153	269	223	127	174
111AIY	0	1	1	110	141	382	184	334	154	317	174	177	172	161	377	153	269	221	127	174
111AIZ	0	1	1	113	141	382	184	334	154	317	174	177	172	161	377	153	269	221	127	174
111AJA	0	1	1	113	141	379	192	325	134	301	153	160	169	161	359	141	275	167	127	178
111AJB	0	7	2	119	141	379	192	325	134	301	153	160	169	161	359	141	275	169	127	178
111AJC	0	2	2	113	141	382	192	349	134	320	159	353	169	161	350	159	275	169	127	178
111AJD	3	16	9	113	145	376	196	343	134	301	159	171	169	161	359	141	275	195	127	178
111AJE	0	2	2	113	141	387	192	352	134	301	184	171	169	161	359	180	275	169	127	186
111AJF	0	2	1	135	155	385	192	352	134	301	159	171	169	161	359	123	275	191	127	178
111AJG	0	1	1	119	138	408	184	358	410	314	159	233	172	161	374	159	277	183	131	176
111AJH	0	1	1	113	148	393	184	370	134	301	159	218	165	157	353	120	275	180	131	176
111AJI	1	2	3	113	148	405	184	334	151	317	171	183	172	161	353	156	267	171	131	207
111AJJ	0	1	1	113	138	396	184	373	206	314	168	183	172	161	377	135	253	231	131	176
111AJK	0	1	1	113	148	390	184	370	188	314	162	218	178	161	374	162	275	180	131	176
111AJL	0	1	1	131	145	382	192	349	134	301	139	171	169	161	359	141	261	169	127	178
111AJM	0	1	1	113	148	393	184	370	188	314	162	194	178	161	374	162	275	180	131	176

Haplotype	Groundnut isolates	Maize isolates	Provinces	AF28	AF13	AF43	AF22	AF31	AF53	AF34	AF42	AF8	AF16	AF54	AF17	AF11	AF66	AF64	AF63	AF55
111AJN	0	1	1	119	148	405	144	312	131	317	146	147	169	161	362	123	269	161	127	178
111AJO	0	4	4	135	155	385	192	352	134	301	159	171	169	161	359	123	275	193	127	180
111AJP	2	6	5	113	138	393	184	364	151	314	159	194	172	161	374	156	253	178	131	180
111AJQ	0	1	1	131	135	393	196	364	134	301	181	215	169	172	359	123	255	169	133	178
111AJR	0	5	2	131	151	385	192	322	134	301	159	171	169	161	359	123	275	225	127	178
111AJS	0	4	3	113	182	393	184	376	144	314	159	177	175	161	379	153	275	176	131	178
111AJT	1	1	2	113	138	390	184	325	197	314	159	186	175	161	359	180	253	195	131	182
111AJU	0	2	1	113	122	376	196	343	134	301	159	171	169	161	359	141	275	217	127	178
111AJV	0	1	1	135	145	379	184	331	163	304	184	191	172	161	362	141	273	233	127	182
111AJW	0	1	1	119	148	385	204	349	134	301	159	174	169	161	359	141	261	174	127	180
111AJX	0	1	1	119	141	385	184	352	257	317	159	242	169	161	377	132	277	211	131	207
111AJY	0	7	4	119	164	399	144	312	131	301	143	168	169	161	371	138	269	163	127	172
111AJZ	0	9	2	113	155	379	192	322	134	301	171	171	169	161	359	138	263	189	127	178
111AKA	0	2	1	113	155	379	192	322	134	301	168	171	169	161	359	138	263	189	127	178
111AKB	0	1	1	110	148	376	196	322	134	301	171	194	169	161	371	150	261	291	127	178
111AKC	0	1	1	113	155	379	192	322	134	301	168	171	169	161	359	135	263	189	127	178
111AKD	0	1	1	113	145	385	192	352	134	298	159	171	169	161	359	141	261	219	127	184
111AKE	0	2	1	113	145	385	192	352	134	301	159	171	169	161	359	141	261	219	127	184
111AKF	0	2	1	113	145	385	192	352	134	301	159	171	169	161	359	144	261	219	127	184
111AKG	0	3	1	131	145	385	192	349	134	301	139	171	169	161	359	138	261	169	127	178
111AKH	0	1	1	113	145	385	192	352	134	301	159	171	169	161	359	144	261	219	127	182
111AKI	0	1	1	113	145	385	192	352	134	301	159	171	169	161	356	144	261	219	127	184
111AKJ	0	1	1	131	138	393	196	361	134	301	181	215	169	172	359	123	255	171	131	178
111AKK	0	1	1	119	145	402	144	312	131	296	146	177	169	161	368	132	269	159	127	174
111AKL	0	1	1	113	158	387	192	315	134	342	159	160	172	161	353	138	263	183	127	176
111AKM	0	7	5	113	155	382	184	370	154	314	162	183	169	161	374	165	275	183	131	182
111AKN	0	1	1	110	141	414	184	358	154	314	162	183	172	161	368	177	255	199	131	178
111AKO	0	1	1	113	141	382	184	334	154	317	178	177	172	161	377	153	269	221	127	172
111AKP	0	1	1	135	141	385	192	346	134	301	159	168	169	161	359	138	261	169	127	178

Haplotype	Groundnut isolates	Maize isolates	Provinces	AF28	AF13	AF43	AF22	AF31	AF53	AF34	AF42	AF8	AF16	AF54	AF17	AF11	AF66	AF64	AF63	AF55
111AKQ	0	1	1	131	148	385	192	352	134	301	171	171	169	161	359	123	261	169	127	180
111AKR	0	1	1	119	128	402	144	312	131	296	146	177	169	161	365	132	269	159	127	174
111AKS	0	1	1	113	158	387	192	315	134	342	159	160	172	161	353	135	263	183	127	178
111AKT	0	1	1	113	158	370	192	325	134	301	178	160	169	161	350	138	263	183	127	184
111AKU	0	3	2	119	141	414	184	352	292	317	159	242	169	161	377	132	277	211	131	207
111AKV	0	1	1	131	148	385	192	352	134	301	159	171	169	161	359	117	261	169	127	180
111AKW	0	1	1	113	141	379	200	322	134	301	181	183	169	161	350	156	265	165	127	180
111AKX	0	1	1	110	138	370	192	325	134	301	159	209	169	161	353	138	261	183	127	176
111AKY	0	5	4	135	141	385	192	346	134	301	159	168	169	161	359	141	261	169	127	178
111AKZ	1	1	2	110	141	382	184	334	154	317	178	177	172	161	377	153	269	221	127	174
111ALA	1	2	3	113	141	370	192	358	134	301	159	160	169	161	353	138	275	163	127	180
111ALB	0	1	1	119	141	414	184	352	213	317	159	236	169	161	377	132	277	209	131	207
111ALC	0	1	1	113	128	387	184	370	134	314	184	174	172	161	353	138	267	243	127	193
111ALD	1	3	4	110	128	387	184	370	134	314	184	174	172	161	368	138	267	243	127	193
111ALE	2	0	2	113	141	370	192	312	134	317	217	160	175	161	359	138	255	169	127	178
111ALF	1	0	1	131	135	379	196	361	134	301	181	215	169	172	359	123	269	161	133	180
111ALG	2	2	3	135	145	385	192	367	134	301	159	160	169	161	362	141	261	169	127	184
111ALH	1	0	1	113	141	379	196	361	134	320	181	209	178	172	359	159	253	227	127	178
111ALI	1	0	1	113	141	385	184	358	154	314	162	183	172	161	371	177	255	199	131	178
111ALJ	1	0	1	131	135	393	196	361	134	301	181	212	169	172	359	123	269	161	133	182
111ALK	1	0	1	119	151	385	192	322	134	301	159	171	169	161	359	123	255	169	127	178
111ALL	1	0	1	110	151	385	196	358	134	301	190	171	169	161	359	141	275	221	127	176
111ALM	1	0	1	113	141	414	184	358	154	314	162	183	172	161	371	177	253	199	131	176
111ALN	1	0	1	113	138	385	184	373	206	314	168	183	172	161	377	135	269	161	131	182
111ALO	1	0	1	113	141	379	196	361	134	320	181	209	178	172	359	159	255	227	129	176
111ALP	1	0	1	131	148	385	192	352	134	301	159	171	169	161	359	123	261	167	127	178
111ALQ	3	2	4	131	151	385	192	322	134	301	159	171	169	161	359	123	275	223	127	178
111ALR	1	0	1	113	145	385	179	322	160	314	171	171	172	161	362	153	261	197	127	170
111ALS	1	1	2	131	135	396	196	364	134	301	181	215	169	172	359	123	257	169	133	178

Haplotype	Groundnut isolates	Maize isolates	Provinces	AF28	AF13	AF43	AF22	AF31	AF53	AF34	AF42	AF8	AF16	AF54	AF17	AF11	AF66	AF64	AF63	AF55
111ALT	1	0	1	113	138	426	184	373	213	314	168	183	172	161	377	135	253	231	131	176
111ALU	1	0	1	119	135	385	192	349	134	301	159	171	169	161	359	141	255	215	127	178
111ALV	1	1	2	135	141	390	200	355	134	301	159	171	169	161	356	141	275	183	127	176
111ALW	1	0	1	135	148	385	196	322	134	320	159	174	169	161	359	135	261	167	127	178
111ALX	1	0	1	113	141	411	184	358	154	314	162	183	172	161	371	177	253	199	131	178
111ALY	1	3	4	131	135	379	196	361	134	301	181	218	169	172	359	123	255	169	133	178
111ALZ	0	1	1	119	141	385	184	352	292	317	159	242	169	161	377	132	277	211	131	207
111AMA	0	1	1	113	141	387	192	364	134	301	159	171	169	161	359	141	275	169	127	178
111AMB	0	3	3	113	145	385	192	364	134	301	159	171	169	161	359	159	261	169	127	178
111AMC	0	14	4	131	151	385	192	322	134	301	159	171	169	161	359	123	275	229	127	178
111AMD	0	3	3	131	151	385	192	322	134	301	159	171	169	161	359	123	275	227	127	178
111AME	0	1	1	113	141	379	196	361	134	320	181	209	178	172	359	159	253	227	129	176
111AMF	0	2	2	113	138	385	184	373	213	314	168	183	172	161	377	135	253	231	131	176
111AMG	0	1	1	110	155	382	184	370	154	314	162	183	169	161	374	165	275	183	131	182
111AMH	0	2	2	113	138	387	184	367	154	314	159	171	169	161	377	168	253	183	131	182
111AMI	0	1	1	131	135	396	196	364	134	301	187	215	169	172	359	123	255	169	133	178
111AMJ	0	5	1	135	158	396	192	315	134	323	159	171	169	161	359	141	261	167	127	184
111AMK	0	1	1	131	135	379	196	325	134	301	181	212	169	172	359	123	255	169	133	178
111AML	0	4	1	135	145	379	184	331	157	304	184	191	172	161	362	141	273	233	127	182
111AMM	0	1	1	131	145	385	192	346	134	320	159	160	169	161	359	141	261	169	127	180
111AMN	0	1	1	113	141	387	184	358	154	314	162	183	172	161	371	177	253	199	131	178
111AMO	0	1	1	119	138	408	184	358	404	314	159	233	172	161	374	159	277	183	131	176
111AMP	0	1	1	113	141	370	192	312	134	326	227	160	175	161	359	138	253	169	127	178
111AMQ	0	1	1	119	138	408	184	358	369	314	159	233	172	161	374	159	277	183	131	176
111AMR	0	3	2	113	148	393	184	370	188	314	162	218	178	161	374	162	275	183	131	176
111AMS	0	1	1	113	148	393	184	370	188	314	162	218	175	161	374	162	275	180	131	176
111AMT	0	1	1	113	148	385	184	373	154	317	181	230	172	161	353	138	271	277	127	191
111AMU	0	1	1	113	128	387	184	370	134	314	184	174	172	161	368	138	267	257	127	193
111AMV	0	1	1	113	141	376	184	379	154	314	162	183	172	161	371	177	255	199	131	178

Haplotype	Groundnut isolates	Maize isolates	Provinces	AF28	AF13	AF43	AF22	AF31	AF53	AF34	AF42	AF8	AF16	AF54	AF17	AF11	AF66	AF64	AF63	AF55
111AMW	0	1	1	119	138	376	184	358	188	314	168	200	172	161	353	162	253	209	131	207
111AMX	0	1	1	113	122	376	196	343	134	301	159	171	169	161	359	141	275	221	127	178
111AMY	0	1	1	113	141	379	184	358	154	314	162	183	172	161	371	177	253	199	131	178
111AMZ	0	3	1	131	145	385	192	349	134	301	139	160	169	161	359	141	261	169	127	178
111ANA	0	9	1	113	141	370	192	352	134	301	159	163	169	161	353	138	275	185	127	180
111ANB	0	1	1	119	138	408	184	358	134	314	159	233	172	161	374	159	277	183	131	176
111ANC	0	1	1	131	135	393	196	361	134	301	181	221	169	172	359	123	255	169	133	178
111AND	0	1	1	119	138	385	192	325	134	301	159	160	169	161	359	144	261	169	127	180
111ANE	0	1	1	119	141	382	179	299	134	310	165	168	178	176	356	144	263	223	131	172
111ANF	0	1	1	119	138	408	184	358	410	314	159	233	172	161	374	159	277	185	131	176
111ANG	1	1	2	113	141	379	192	315	134	320	159	171	169	161	359	159	279	169	129	182
111ANH	0	2	2	119	138	408	184	358	414	314	159	233	172	161	374	159	277	183	131	176
111ANI	0	1	1	131	151	385	192	322	134	301	159	171	169	161	359	123	275	231	127	178
111ANJ	0	1	1	113	148	385	176	370	157	314	184	203	172	161	362	138	267	174	127	195
111ANK	0	1	1	110	141	379	196	361	134	320	181	209	178	172	359	159	255	227	129	178
111ANL	0	1	1	119	138	408	184	358	404	314	156	233	172	161	374	159	277	183	131	176
111ANM	0	1	1	113	167	393	184	367	151	314	159	183	175	161	359	165	261	201	129	180
111ANN	0	7	1	113	167	393	184	367	151	314	159	183	175	161	359	165	261	201	127	180
111ANO	0	1	1	113	167	393	184	367	151	314	159	183	175	161	359	165	261	201	127	178
111ANP	0	1	1	119	128	379	144	312	131	314	146	183	169	161	362	138	269	163	125	172
111ANQ	0	1	1	113	148	393	184	370	188	314	162	218	178	161	374	162	275	180	131	174
111ANR	0	1	1	131	138	385	192	349	134	301	159	174	175	161	359	123	261	174	127	184
111ANS	0	1	1	113	145	387	192	346	134	301	159	171	169	161	359	141	273	169	127	178
111ANT	0	2	1	119	128	379	144	312	131	296	146	183	169	161	362	138	269	163	125	172
111ANU	0	1	1	110	145	376	192	322	134	301	159	171	169	161	359	141	261	169	127	178
111ANV	0	1	1	135	145	379	184	331	157	304	184	191	172	161	362	141	273	235	127	182
111ANW	0	1	1	131	135	396	196	364	134	301	181	215	169	172	359	123	255	169	133	178
111ANX	0	1	1	113	122	385	184	337	151	317	171	194	172	161	359	135	267	207	127	207
111ANY	0	2	2	135	148	387	179	331	163	317	162	168	169	161	365	138	271	195	131	195

Haplotype	Groundnut isolates	Maize isolates	Provinces	AF28	AF13	AF43	AF22	AF31	AF53	AF34	AF42	AF8	AF16	AF54	AF17	AF11	AF66	AF64	AF63	AF55
111ANZ	0	2	2	131	145	385	192	349	134	301	139	171	169	161	359	141	261	167	127	178
111AOA	0	1	1	113	182	396	184	376	144	314	159	177	175	161	379	153	275	176	131	178
111AOB	0	4	3	119	138	399	184	361	160	314	168	209	169	161	374	159	277	217	131	176
111AOC	0	1	1	113	141	414	184	361	154	314	162	183	172	161	371	177	253	199	131	178
111AOD	0	1	1	113	158	382	184	328	157	317	153	239	172	161	359	138	267	207	127	197
111AOE	0	1	1	119	138	408	184	358	401	314	159	233	172	161	374	159	277	183	131	176
111AOF	0	1	1	113	135	379	196	337	134	301	181	212	169	172	359	123	255	169	133	178
111AOG	0	3	3	113	141	379	196	361	134	320	181	209	178	172	359	159	253	225	129	178
111AOH	0	1	1	113	141	385	184	358	154	314	162	183	172	161	359	174	255	199	131	178
111AOI	0	1	1	113	141	370	192	355	134	301	159	160	169	161	353	138	275	163	127	180
111AOJ	0	1	1	113	141	414	184	361	154	314	162	183	172	161	371	177	255	199	131	178
111AOK	1	1	2	113	138	393	184	364	151	314	159	194	172	161	374	156	255	178	131	180
111AOL	0	1	1	113	138	402	184	373	213	314	168	183	172	161	377	135	253	231	131	176
111AOM	0	1	1	113	200	390	184	370	134	314	184	174	172	161	368	138	267	245	127	193
111AON	0	2	2	113	138	393	184	364	151	314	159	194	172	161	374	156	253	180	131	180
111AOO	0	1	1	119	164	399	144	312	131	307	143	168	169	161	371	138	269	163	127	172
111AOP	0	2	2	131	135	393	196	361	134	301	178	212	169	172	359	123	255	169	133	178
111AOQ	0	1	1	131	145	385	192	349	134	301	139	171	169	172	359	123	255	169	133	178
111AOR	1	0	1	113	141	390	184	358	154	314	162	186	172	161	371	177	253	199	131	178
111AOS	1	0	1	135	155	385	192	352	134	301	159	171	169	161	359	141	261	169	127	178
111AOT	1	0	1	131	145	385	192	349	134	301	139	171	165	161	359	141	261	169	127	178
111AOU	1	0	1	113	141	390	184	358	154	314	162	183	172	161	371	177	253	199	131	178
111AOV	1	0	1	135	155	390	196	349	134	301	159	171	172	161	365	126	255	169	127	178
111AOW	1	0	1	128	141	414	184	358	154	314	162	183	172	161	371	177	255	199	131	178
111AOX	1	0	1	113	141	385	184	367	188	310	174	168	175	161	359	153	271	215	131	180
111AOY	1	0	1	113	158	385	192	315	154	317	162	186	169	161	359	141	261	169	127	184
111AOZ	1	0	1	135	141	385	192	346	134	301	159	168	169	161	359	144	261	169	127	178
111APA	1	2	2	113	155	376	192	325	134	301	181	189	172	161	353	138	263	183	127	180
111APB	1	1	2	113	145	376	184	334	151	314	165	183	175	161	359	150	271	183	131	186

Haplotype	Groundnut isolates	Maize isolates	Provinces	AF28	AF13	AF43	AF22	AF31	AF53	AF34	AF42	AF8	AF16	AF54	AF17	AF11	AF66	AF64	AF63	AF55
111APC	1	0	1	135	161	385	192	315	134	323	162	171	169	161	359	141	261	169	127	184
111APD	1	0	1	113	141	414	184	358	154	314	162	183	172	161	371	177	255	201	131	178
111APE	1	0	1	113	138	390	184	325	197	314	159	186	175	161	359	180	255	195	131	182
111APF	1	0	1	119	148	405	144	312	131	296	146	147	169	161	362	123	271	161	127	178
111APG	2	0	1	135	148	385	196	322	134	320	159	174	169	161	359	141	261	169	127	178
111APH	0	1	1	113	141	370	184	358	154	314	162	183	172	161	371	177	253	199	131	178
111API	0	1	1	131	135	379	196	337	134	301	181	212	169	172	359	123	248	169	133	178
111APJ	0	2	2	110	148	393	184	370	188	314	162	218	178	161	374	162	275	180	131	176
111APK	0	3	2	113	138	405	184	340	206	314	168	183	172	161	374	135	277	183	131	176
111APL	0	1	1	119	128	405	144	312	131	296	146	177	169	161	368	132	269	159	127	174
111APM	0	2	2	131	135	393	196	361	134	301	181	212	169	172	359	123	257	169	133	178
111APN	0	1	1	113	122	376	196	343	134	301	159	171	169	161	359	141	275	215	127	178
111APO	0	1	1	113	141	408	192	312	134	326	146	160	175	161	359	138	255	169	127	178
111APP	0	1	1	113	145	387	192	364	134	301	159	171	169	161	359	123	261	169	127	178
111APQ	0	3	3	113	148	393	184	370	197	314	159	218	178	161	374	162	275	180	131	176
111APR	0	2	2	119	128	414	144	312	131	301	146	174	169	161	368	132	269	159	127	174
111APS	0	1	1	113	145	376	196	343	134	301	159	177	169	161	359	141	275	195	127	178
111APT	0	6	3	113	145	376	184	334	151	314	165	186	175	161	393	150	271	183	131	186
111APU	0	1	1	135	155	385	192	352	134	298	159	171	169	161	359	123	275	191	127	180
111APV	0	8	3	119	128	402	144	312	131	301	146	177	169	161	368	132	269	159	127	174
111APW	0	1	1	131	148	385	192	352	134	304	156	171	169	161	359	123	261	169	127	180
111APX	0	1	1	113	151	385	196	358	134	301	190	171	169	161	359	141	275	223	127	178
111APY	0	7	1	113	141	382	192	299	134	317	199	186	169	161	374	159	275	185	133	180
111APZ	0	1	1	113	148	393	184	370	188	314	159	218	178	161	374	162	275	180	131	176
111AQA	0	1	1	119	141	414	184	352	295	317	156	242	169	161	377	132	277	211	131	207
111AQB	0	1	1	113	145	376	196	343	134	304	159	171	165	161	359	141	275	195	127	178
111AQC	0	1	1	119	128	402	144	312	131	296	143	177	169	161	368	132	269	159	127	174
111AQD	0	2	2	142	138	379	196	361	134	320	181	218	169	172	371	141	263	185	133	180
111AQE	0	1	1	113	138	393	184	364	151	314	162	194	172	161	374	156	253	178	131	180

Haplotype	Groundnut isolates	Maize isolates	Provinces	AF28	AF13	AF43	AF22	AF31	AF53	AF34	AF42	AF8	AF16	AF54	AF17	AF11	AF66	AF64	AF63	AF55
111AQF	0	1	1	131	135	393	196	361	134	304	178	215	169	172	359	123	255	169	133	178
111AQG	0	1	1	135	155	385	192	352	134	304	159	171	169	161	359	123	275	191	127	180
111AQH	0	1	1	113	141	379	196	361	134	320	184	209	178	172	359	159	253	227	129	178
111AQI	0	1	1	113	141	382	184	358	154	314	162	183	172	161	371	177	253	199	131	178
111AQJ	0	1	1	113	161	385	184	334	151	314	165	191	175	161	377	156	275	180	131	178
111AQK	0	1	1	113	173	393	184	334	163	314	181	177	175	161	374	180	261	169	127	180
111AQL	0	1	1	131	148	385	192	352	134	301	159	171	169	161	359	123	267	243	127	193
111AQM	0	1	1	113	128	387	184	370	134	314	184	174	172	161	368	138	275	180	131	176
111AQN	0	1	1	135	155	385	192	352	134	301	159	171	169	161	359	123	279	169	129	184
111AQO	0	1	1	113	141	379	192	315	134	320	159	171	169	161	359	159	277	178	131	178
111AQP	0	5	3	131	158	385	192	315	134	320	162	171	169	161	359	141	261	169	127	184
111AQQ	0	1	1	135	155	385	192	352	134	301	156	171	169	161	359	123	275	191	127	180
111AQR	0	1	1	113	138	387	184	325	206	314	159	189	175	161	359	177	253	203	131	184
111AQS	0	2	1	113	145	376	184	334	151	314	165	183	175	161	396	150	271	183	131	186
111AQT	0	4	1	135	141	390	200	376	134	301	159	174	169	161	356	150	275	183	127	176
111AQU	0	1	1	113	145	385	192	364	134	301	159	171	169	161	359	159	261	169	133	178
111AQV	0	1	1	113	141	379	196	361	134	320	181	209	169	172	359	123	253	227	129	178
111AQW	0	3	1	131	135	393	196	361	134	301	181	212	178	161	374	162	255	169	133	178
111AQX	0	1	1	131	135	393	196	364	134	301	181	212	172	161	353	138	255	169	133	178
111AQY	0	1	1	131	135	393	196	361	134	301	181	212	169	161	359	123	255	169	133	178
111AQZ	0	1	1	131	135	393	196	361	134	301	181	212	178	172	359	159	255	169	133	178
111ARA	0	3	1	113	148	393	184	370	197	314	162	218	169	172	359	123	275	180	131	176
111ARB	0	1	1	113	141	373	192	334	134	342	178	171	169	172	359	123	253	183	127	178
111ARC	0	1	1	131	148	385	192	352	134	301	159	171	169	172	359	123	261	169	127	180
111ARD	0	1	1	125	141	390	184	370	263	314	165	180	172	161	374	165	277	183	127	176
111ARE	0	1	1	113	145	387	192	364	134	301	159	171	169	161	359	141	273	169	127	178
111ARF	0	1	1	113	173	393	184	334	163	314	181	177	175	161	374	180	275	180	131	178
111ARG	0	1	1	113	141	370	192	352	134	301	153	160	169	161	353	138	275	185	127	180
111ARH	0	1	1	138	176	370	179	334	144	320	190	189	213	161	356	218	273	171	127	172

Haplotype	Groundnut isolates	Maize isolates	Provinces	AF28	AF13	AF43	AF22	AF31	AF53	AF34	AF42	AF8	AF16	AF54	AF17	AF11	AF66	AF64	AF63	AF55
111ARI	0	1	1	113	145	376	200	343	134	301	159	174	169	161	359	141	275	195	127	178
111ARJ	0	1	1	113	145	376	200	343	134	301	159	171	169	161	359	141	275	197	127	178
111ARK	0	1	1	113	141	379	196	361	134	320	181	209	178	172	359	159	253	231	129	178
111ARL	0	1	1	119	128	385	144	312	131	296	146	174	169	161	368	132	269	159	127	174
111ARM	0	1	1	131	135	393	196	361	134	301	178	212	169	161	359	141	275	197	127	178
111ARN	0	1	1	131	135	393	196	361	134	301	159	171	169	161	359	141	257	169	133	178
111ARO	0	1	1	138	145	385	192	322	134	301	181	212	169	172	359	123	261	169	127	178
111ARP	0	1	1	113	148	393	184	370	134	301	159	212	169	172	359	123	275	180	131	176
111ARQ	0	1	1	113	145	376	200	343	134	301	156	171	169	161	359	141	275	195	127	178
111ARR	0	1	1	113	155	382	184	370	154	314	162	183	169	161	374	165	275	183	131	180
111ARS	0	1	1	110	122	376	196	343	134	301	159	171	169	161	359	141	275	215	127	178
111ART	0	1	1	131	148	385	192	352	134	301	159	171	169	161	359	123	275	183	131	182
111ARU	0	1	1	131	145	385	192	349	154	314	162	183	169	161	374	165	261	169	127	180
111ARV	0	1	1	131	145	385	192	346	134	301	159	171	169	161	359	123	261	169	127	178
111ARW	0	1	1	119	128	408	144	312	134	301	139	171	169	161	359	141	269	159	127	174
111ARX	0	1	1	119	148	405	144	312	134	301	139	171	169	161	362	123	269	159	127	174
111ARY	0	1	1	113	141	414	184	358	154	310	162	183	172	161	371	177	269	161	127	178
111ARZ	0	1	1	113	141	414	184	358	131	296	146	147	169	161	362	123	255	199	131	178
111ASA	0	1	1	119	164	399	144	312	131	296	143	168	172	161	371	177	255	199	131	178
111ASB	0	1	1	113	141	379	196	361	154	314	162	183	178	172	359	159	269	163	127	172
111ASC	0	1	1	119	141	376	196	322	134	301	187	183	169	161	353	126	263	237	127	176
111ASD	0	1	1	119	148	405	144	312	131	301	143	168	169	161	371	138	255	227	129	178
111ASE	0	1	1	113	141	414	184	358	154	320	181	209	178	172	359	159	253	199	131	178
111ASF	0	1	1	135	155	385	192	352	131	301	146	147	169	161	362	123	255	199	131	178
111ASG	0	1	1	119	128	408	144	312	131	301	146	180	169	161	368	132	269	159	127	174
111ASH	0	1	1	113	145	390	192	373	134	301	159	171	169	161	377	159	273	213	127	176
111ASI	0	1	1	135	145	385	192	376	134	326	159	171	169	161	356	141	261	183	127	178
111ASJ	0	1	1	119	128	373	144	312	131	301	146	174	169	161	368	132	269	159	127	174
111ASK	1	0	1	131	135	379	196	361	134	301	181	215	169	172	359	123	257	169	133	178

Haplotype	Groundnut isolates	Maize isolates	Provinces	AF28	AF13	AF43	AF22	AF31	AF53	AF34	AF42	AF8	AF16	AF54	AF17	AF11	AF66	AF64	AF63	AF55
111ASL	1	0	1	135	141	379	196	315	134	323	159	353	169	172	359	159	263	169	133	180
111ASM	0	1	1	131	135	379	196	337	134	304	178	212	169	172	359	123	255	169	133	178
111ASN	0	1	1	113	141	379	196	361	134	320	181	209	178	172	359	159	255	229	129	178
111ASO	0	1	1	131	151	385	192	322	134	304	156	171	169	161	359	123	275	229	127	178
111ASP	0	1	1	138	164	393	184	370	169	317	184	218	172	161	353	138	267	176	127	197
111ASQ	0	1	1	113	155	382	184	367	154	314	162	183	169	161	374	165	275	183	131	182
111ASR	0	1	1	113	138	396	184	334	151	314	168	180	175	161	374	162	255	183	131	182
111ASS	0	1	1	113	151	385	196	358	134	301	190	171	169	169	359	141	275	221	127	178
111AST	0	1	1	135	141	390	200	376	134	301	156	174	169	161	353	150	275	183	127	176
111ASU	0	2	1	131	135	393	196	361	134	304	181	212	169	172	359	123	255	169	133	178
111ASV	0	1	1	131	135	393	196	361	134	304	178	212	169	172	359	123	255	169	133	178
111ASW	0	1	1	110	141	385	176	349	154	317	159	197	172	161	362	144	273	203	127	199
111ASX	0	1	1	135	148	390	179	331	163	317	162	168	169	161	365	138	271	195	131	195
111ASY	0	2	1	110	122	385	184	337	134	317	205	206	172	161	359	135	267	183	127	197
111ASZ	0	1	1	113	122	385	213	337	134	317	205	206	172	161	359	135	267	183	127	197
111ATA	0	1	1	113	138	402	184	373	213	314	168	183	172	161	377	135	253	229	131	176
111ATB	0	1	1	113	138	385	184	373	213	314	168	183	172	161	377	135	253	229	131	176
111ATC	0	1	1	113	122	385	184	337	134	317	205	206	172	161	359	135	267	183	127	199
111ATD	0	4	1	113	145	376	184	334	151	314	165	183	175	161	393	150	271	185	131	186
111ATE	0	1	1	113	145	376	184	334	151	314	165	183	175	161	365	150	271	185	131	186
111ATF	0	1	1	113	145	376	184	334	151	314	165	183	175	161	353	150	271	185	131	186
111ATG	0	1	1	113	145	376	184	334	151	314	165	183	175	161	356	150	271	185	131	186
111ATH	16	0	3	113	145	385	192	349	134	301	159	171	169	161	359	141	261	199	127	184
111ATI	1	0	1	131	138	376	192	322	134	301	159	171	169	161	359	141	275	169	131	180
111ATJ	1	0	1	119	145	402	179	331	144	317	162	168	169	169	353	144	275	185	131	195
111ATK	1	1	2	113	145	385	192	352	134	301	159	174	169	161	359	123	273	169	127	180
111ATL	4	0	2	135	145	387	192	352	134	301	159	174	169	161	356	141	261	201	127	178
111ATM	0	0	1	113	145	385	192	349	134	301	159	171	169	161	359	141	261	201	127	186
111ATN	0	0	1	131	138	376	192	322	134	301	159	171	169	161	359	141	275	169	127	180

Haplotype	Groundnut isolates	Maize isolates	Provinces	AF28	AF13	AF43	AF22	AF31	AF53	AF34	AF42	AF8	AF16	AF54	AF17	AF11	AF66	AF64	AF63	AF55
111ATO	3	0	1	113	138	385	192	315	134	320	159	171	169	161	350	159	263	183	127	184
111ATP	1	0	1	131	135	379	196	361	134	301	181	215	169	172	359	123	255	167	133	176
111ATQ	0	1	1	113	145	379	192	352	134	301	159	174	169	161	359	123	273	169	127	178
111ATR	0	1	1	113	141	379	176	346	169	314	162	177	172	161	362	159	275	213	127	186
111ATS	0	1	1	113	145	379	176	346	169	314	162	177	172	161	362	159	275	213	127	186
111ATT	0	1	1	116	145	385	176	346	169	314	162	177	172	161	362	159	275	213	127	186
111ATU	0	1	1	113	145	379	176	346	169	314	162	177	169	145	362	159	275	213	127	186
111ATV	0	1	1	113	138	376	200	334	134	301	178	163	169	161	371	156	275	169	127	178
111ATW	0	3	1	110	138	376	200	334	134	301	178	163	169	161	371	156	275	169	127	178
111ATX	0	1	1	131	145	385	192	349	134	301	139	171	169	161	359	141	261	167	127	176
111ATY	0	1	1	113	135	376	200	334	134	301	178	163	169	161	371	153	275	171	127	178
111ATZ	0	2	1	138	155	382	176	355	147	301	156	174	175	161	374	153	275	183	127	214
111AUA	0	2	1	138	155	382	176	355	147	301	156	174	175	161	374	153	275	183	127	212
111AUB	0	1	1	138	151	382	176	355	147	301	156	174	175	161	374	153	275	183	127	214
111AUC	0	1	1	113	138	370	184	358	154	314	162	183	172	161	371	177	253	199	131	176
111AUD	0	1	1	113	138	379	196	361	134	320	181	209	178	172	359	159	253	225	127	178
111AUE	0	1	1	113	138	379	196	361	134	320	181	209	178	172	359	159	253	227	129	178
111AUF	0	1	1	113	141	414	184	358	154	314	162	183	178	172	359	174	253	199	131	178
111AUG	0	1	1	113	135	387	184	367	154	314	159	171	169	161	377	165	253	180	131	182
111AUH	0	2	2	135	151	385	192	352	134	301	159	171	169	161	359	123	275	189	127	180
111AUI	0	1	1	131	135	385	192	312	134	320	159	215	169	161	359	123	255	169	133	178
111AUJ	0	1	1	113	141	385	184	358	154	314	162	183	172	161	371	177	253	199	131	178
111AUK	0	1	1	131	151	385	192	322	134	301	162	171	169	161	359	123	275	223	127	178
111AUL	0	1	1	113	141	408	192	312	134	304	217	160	175	161	359	138	255	169	127	178
111AUM	0	1	1	131	135	390	173	337	134	301	193	144	203	172	356	150	269	239	127	172
111AUN	0	3	2	135	141	390	200	331	134	301	159	171	169	161	356	141	275	183	127	176
111AUO	0	2	2	131	161	387	192	358	134	301	181	160	169	161	359	141	261	169	127	180
111AUP	0	5	4	113	141	408	192	312	134	326	217	160	175	161	359	138	253	169	127	178
111AUQ	0	11	2	113	138	376	200	334	134	301	178	163	169	161	371	156	275	171	127	180

Haplotype	Groundnut isolates	Maize isolates	Provinces	AF28	AF13	AF43	AF22	AF31	AF53	AF34	AF42	AF8	AF16	AF54	AF17	AF11	AF66	AF64	AF63	AF55
111BOC	0	1	1	119	128	408	144	312	131	296	150	174	169	161	362	132	269	161	127	174
111DYT	0	1	1	113	161	387	188	346	157	301	181	194	172	161	365	138	267	163	127	191
111GTT	0	1	1	113	167	393	184	367	151	314	159	183	175	161	359	165	261	201	121	180
111GTU	0	1	1	119	145	405	144	312	131	296	146	147	169	161	362	123	269	161	127	178
111GTV	0	2	2	131	135	390	173	337	134	301	193	144	203	172	356	150	269	239	127	174
111GTW	0	1	1	113	141	370	192	312	134	317	217	160	175	161	359	138	253	169	127	178
111GTX	0	1	1	135	125	387	179	379	144	301	220	194	200	172	350	209	269	183	131	189

*Allele sizes indicate amplicon size in base pairs as called on an ABI 3730 DNA Analyzer with the LIZ500 standard (Applied Biosystems).
