# Supplementary material for: Aflatoxin contamination of maize and groundnut in Burundi: Distribution of contamination, identification of causal agents and potential biocontrol genotypes of Aspergillus flavus
Source: Front Microbiol. 2023 Mar 13;14:1106543. doi: 10.3389/fmicb.2023.1106543 (PMC10093718; doi:10.3389/fmicb.2023.1106543)
Supplement: Supplementary file 2 [file Table_2.pdf]

Supplementary Table 2. Results of AMOVA comparing isolates from maize and groundnut in Burundi as calculated by Arlequin 3.5.2.2 (Excoffier and Lischer, 2010).

Source of variation	Degrees of freedom	Sum of squares	Variance components	Percentage of variation
Among crops	1	7.3	0.004	0.06
Within crops	1,088	6,774.3	6.226	99.94
Total	1,089	6,781.6	6.230	
Fixation index	F_{ST} = 0.00063			
