# Supplementary material for: Aflatoxin contamination of maize and groundnut in Burundi: Distribution of contamination, identification of causal agents and potential biocontrol genotypes of Aspergillus flavus
Source: Front Microbiol. 2023 Mar 13;14:1106543. doi: 10.3389/fmicb.2023.1106543 (PMC10093718; doi:10.3389/fmicb.2023.1106543)
Supplement: Supplementary file 3 [file Table_3.pdf]

Supplementary Table 3. Number of provinces the 20 most common haplotypes were found.

Haplotype	Sample corrected count	Number of provinces
111AGS	58	15
111AHG	40	8
111AII	34	8
111AHF	33	9
111AIA	32	11
111ABU	28	12
111AIO	25	12
111AIB	25	12
111AGV	24	8
111AHN	23	9
111AHM	23	11
111AIV	22	11
111AHO	22	11
111AHH	22	8
111AHL	19	11
111AHQ	18	10
111AHC	18	7
111AGW	18	8
111AJD	15	9
111AHB	15	7
Average		9.9
