# Supplementary material for: Aflatoxin contamination of maize and groundnut in Burundi: Distribution of contamination, identification of causal agents and potential biocontrol genotypes of Aspergillus flavus
Source: Front Microbiol. 2023 Mar 13;14:1106543. doi: 10.3389/fmicb.2023.1106543 (PMC10093718; doi:10.3389/fmicb.2023.1106543)
Supplement: Supplementary file 4 [file Table_4.pdf]

Supplementary Table 4. Results of an AMOVA analyzing diversity by province in Burundi as calculated by Arlequin 3.5.2.2 (Excoffier and Lischer, 2010).

Source of variation	Degrees of freedom	Sum of squares	Variance component	Percentage of variation
Among provinces	15	139.3	0.047	0.76
Within provinces	1,074	6,641.9	6.184	99.24
Total	1,089	6,781.2	6.231	
Fixation index	F_{ST} = 0.00756			
